# The transcription factor Pitx2 positions the embryonic axis and regulates twinning

**DOI:** 10.7554/eLife.03743

**Published:** 2014-12-12

**Authors:** Angela Torlopp, Mohsin A F Khan, Nidia M M Oliveira, Ingrid Lekk, Luz Mayela Soto-Jiménez, Alona Sosinsky, Claudio D Stern

**Affiliations:** 1 Department of Cell and Developmental Biology, University College London, London, United Kingdom; 2 Programa de Ciencias Genómicas, Universidad Nacional Autónoma de México, Morelos, Mexico; 3 Institute of Structural and Molecular Biology, Birkbeck College, University of London, London, United Kingdom; California Institute of Technology, United States

**Keywords:** gastrulation, primitive streak, embryonic axis, embryo polarity, chicken

## Abstract

Embryonic polarity of invertebrates, amphibians and fish is specified largely by maternal determinants, which fixes cell fates early in development. In contrast, amniote embryos remain plastic and can form multiple individuals until gastrulation. How is their polarity determined? In the chick embryo, the earliest known factor is cVg1 (homologous to mammalian growth differentiation factor 1, GDF1), a transforming growth factor beta (TGFβ) signal expressed posteriorly before gastrulation. A molecular screen to find upstream regulators of cVg1 in normal embryos and in embryos manipulated to form twins now uncovers the transcription factor Pitx2 as a candidate. We show that Pitx2 is essential for axis formation, and that it acts as a direct regulator of cVg1 expression by binding to enhancers within neighbouring genes. Pitx2, Vg1/GDF1 and Nodal are also key actors in left–right asymmetry, suggesting that the same ancient polarity determination mechanism has been co-opted to different functions during evolution.

**DOI:** http://dx.doi.org/10.7554/eLife.03743.001

## eLife digest

In warm-blooded animals, including chickens and humans, a single embryo can give rise to several separate individuals (identical twins). Some species of armadillos routinely give birth to quadruplets in this way—and in experiments, up to eight identical chick embryos can be produced by cutting one embryo into smaller pieces (a type of ‘experimental twinning’). This ability of a developing embryo to subdivide into separate individuals ends when the embryo starts to form its first midline structure, called the ‘primitive streak’. This is the first line of symmetry and defines where the head–tail axis will later develop.

The steps that establish the axes of the embryo in birds and mammals, and the factors that prevent further splitting of the embryo to form twins after this point, are only just beginning to be understood. In chick embryos, the production of a protein called cVg1 is the first known step and precedes the development of a line of symmetry. A similar protein is produced in mammalian embryos and both proteins are members of an important family of signalling proteins.

Now, Torlopp, Khan et al. have used a combination of techniques to search for other proteins that that control the production of the cVg1 protein. Genes that are active in the region of the embryo that will express cVg1 later in development were identified, both in normal embryos and during the process of experimental twinning. This search revealed Pitx2 as a protein that acts to switch on the expression of the gene that encodes cVg1. When the Pitx2 protein is removed, the embryonic axis forms from the opposite side.

Next, Torlopp, Khan et al. searched the chicken genome to identify stretches of DNA around the cVg1 gene where proteins that regulate gene expression might bind. Six potential sites were found, including four to which Pitx2 can bind. Further experiments confirmed that two of these regulatory sequences encourage the expression of the cVg1 gene at its correct position in the embryo.

Pitx2 and related proteins were known to be involved with the development of left–right symmetry later in development; the findings of Torlopp, Khan et al. reveal, unexpectedly, that these proteins are also involved in first establishing the position at which the midline of the embryo will arise. It remains unclear what prevents most embryos from forming twins. But Torlopp, Khan et al.'s findings could help to explain some strange observations, made long ago, about left–right asymmetry in identical twins. For example, they could help explain why one of the twins in an identical twin pair is more likely to be left-handed than an individual in the general population, and why the direction of whorls of hair on the back of the head is often mirrored between identical twins.

**DOI:** http://dx.doi.org/10.7554/eLife.03743.002

## Introduction

In most invertebrates and anamniote vertebrates (fishes and amphibians), embryonic polarity is first established by localisation of maternal determinants in the cytoplasm and/or cortex of the fertilised egg. This generates differences between the blastomeres that will form by cell division from the egg, and which will culminate in specifying the orientation of the embryonic axes (Wilson, 1898). Separation of the first two blastomeres can lead to twinning: the formation of genetically identical, complete individuals (Driesch, 1892). Separation of blastomeres after the four-cell stage, however, does not generate twins; in most cases it interferes with development of even a single embryo owing to the removal of important determinants that have by then segregated to different cells. This is known as the *mosaic* mode of development. Among the vertebrates, amniotes (birds and many mammals, and possibly also reptiles) have a remarkably extended capacity to give rise to twins. Some species of the armadillo genus Dasypus generate quadruplets or octuplets from a single fertilisation event, as a result of two or more sequential ‘splitting’ events of the embryo at a stage when it is already highly multicellular (Newman and Patterson, 1910; Loughry et al., 1998; Enders, 2002; Eakin and Behringer, 2004). Conjoined (‘Siamese’) twins occur in mammals including humans (Chai and Crary, 1971; Vanderzon et al., 1998; Kaufman, 2004) and are also seen in reptiles (Cunningham, 1937) and birds (Ulshafer and Clavert, 1979); most of these are thought to arise from splitting of the embryo relatively late in development (Kaufman, 2004). Perhaps the most dramatic example is seen in the chick, where cutting an embryo into fragments at the blastoderm stage (when the embryo contains as many as 20,000–50,000 cells) can lead to each fragment generating a complete embryo; up to eight embryos have been generated from a single blastoderm by experimental splitting, right up to the time of appearance of the primitive streak (Lutz, 1949; Spratt and Haas, 1960). The ability of higher vertebrate embryos to retain a *regulative* model of development until such a late stage strongly suggests that localisation of maternally inherited determinants is not an essential component of the mechanisms specifying embryo polarity (Stern and Downs, 2012). Moreover, since a single blastoderm can generate multiple embryos, mechanisms must exist that suppress this ability in regions of the embryo that do not normally initiate axis formation (Bertocchini and Stern, 2002; Bertocchini et al., 2004).

In chick embryos, the earliest symmetry breaking event known is the localised expression of *cVg1*, the chick orthologue of mammalian growth differentiation factor 1 (*GDF1*)—a member of the transforming growth factor beta (TGFβ) superfamily of secreted proteins—encoding a Nodal/Activin-type molecule that signals through Smad2/3 (Weeks and Melton, 1987; Thomsen and Melton, 1993; Kessler and Melton, 1995; Seleiro et al., 1996; Shah et al., 1997; Kessler, 2004; Birsoy et al., 2006; Chen et al., 2006; Andersson et al., 2007). Before primitive streak stages, *cVg1* is expressed in the posterior marginal zone (PMZ), an extraembryonic region adjacent to where the primitive streak will form; misexpression of *cVg1* in other (anterior or lateral) parts of the marginal zone is sufficient to induce a complete axis from adjacent embryonic cells (Seleiro et al., 1996; Shah et al., 1997; Skromne and Stern, 2001, 2002). The mechanisms that position *cVg1* in the PMZ are unknown. Moreover, when a blastoderm is cut in half at right angles to the future primitive streak axis, *cVg1* expression spontaneously initiates in the marginal zone adjacent to the cut edge, in either the right or left side at equal frequency, foreshadowing the appearance of the primitive streak a few hours later (Bertocchini et al., 2004). This observation shows that the mechanisms that position *cVg1* are active in the blastoderm stage embryo. Here we take advantage of these observations to design a molecular screen for new genes involved in the earliest stages of specifying embryo polarity; together with bioinformatic analysis and embryological experiments we identify the transcription factor *Pitx2* as a direct and essential regulator of *cVg1* expression both during normal development and in embryonic regulation (induced twinning).

## Results

### A molecular screen to identify upstream regulators of cVg1 uncovers Pitx2

**Figure 1.**
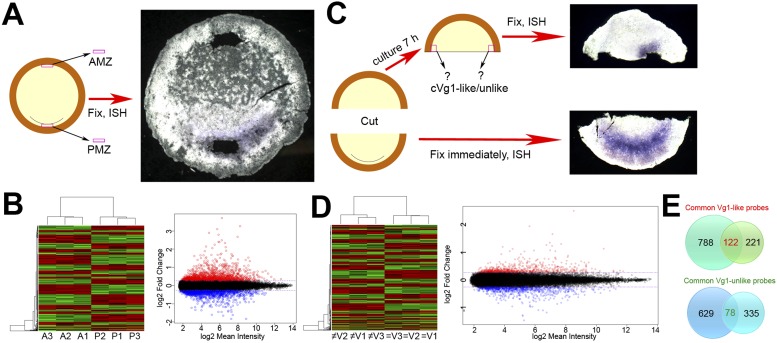
Microarray screens for upstream regulators of cVg1. (**A**) Diagram of the first screen: the posterior marginal zone (PMZ) and anterior marginal zone (AMZ) were dissected from embryos at stage XI–XII; the remaining embryo was then fixed and stained for *cVg1* by in situ hybridisation (ISH) to confirm that the explants had been obtained from the correct regions. This was done from 40 embryos for each of three biological replicates, which were then run on microarrays. The diagram is accompanied by an example of an embryo after ISH. All 120 embryos are shown in Figure 1—figure supplement 1. (**B**) Hierarchical clustering of differentially expressed genes for this experiment, and a plot of where *cVg1*-like probes (enriched in PMZ) are displayed in red and *cVg1*-unlike (‘downregulated’) probes shown in green across triplicate samples (A1–A3 for AMZ, P1–P3 for PMZ). The scatter plot relates normalised log_2_ mean signal intensities and log_2_ fold changes of probes from both samples (AMZ and PMZ). Probes identified as upregulated in the PMZ with a log_2_ fold change cut-off of 0.263 (linear fold change 1.2) are displayed in red and those identified as downregulated in the PMZ with the same cut-off are displayed in blue. (**C**) Diagram of the second screen. An embryo at stage XI–XII was cut in half at a right angle to the future midline; the posterior half was fixed for ISH with *cVg1* to confirm the orientation (an example is shown), and the isolated anterior half cultured for 7 hr. At this point, a small explant was dissected from the marginal zone adjacent to the left and right side of the cut, and the remaining anterior half-embryo fixed for ISH with *cVg1* (an example is shown). This allowed identification of the ‘*cVg1*-like’ and ‘*cVg1*-unlike’ explants, which were then pooled appropriately. This was done for 70 embryos for each of three biological replicates; all 210 posterior and anterior fragments are shown in Figure 1—figure supplement 2 after ISH for *cVg1*. (**D**) Hierarchical clustering of the probes expressed differentially in this assay, and corresponding scatter plot; details similar to (**B**) ≠V1, ≠V2, and ≠V3 correspond to each of the triplicate samples that do not express *cVg1* and = V1, =V2, and = V3 correspond to explants that express *cVg1*. (**E**) Venn diagrams showing the intersection of upregulated and downregulated probes common to both the PMZ and isolated anterior cut halves. A total of 122 upregulated probes and 78 downregulated probes were found to be common in both experiments using both p value and fold change as the criteria. The complete dataset has been submitted to ArrayExpress where it has been assigned the Accession number E-MTAB-3116. **DOI:** http://dx.doi.org/10.7554/eLife.03743.003

To search for putative upstream regulators of *cVg1*, we took advantage of two of its properties: that it is expressed in the PMZ at early stages of development and that when a blastoderm is cut in half at right angles to the axis of the future primitive streak, *cVg1* expression is initiated stochastically on either the left or the right corner (adjacent to the cut edge) of the isolated anterior half (Bertocchini et al., 2004). We therefore performed two screens. First, we dissected the PMZ and an equivalent anterior explant (anterior marginal zone, AMZ) from 40 embryos (in triplicate) and analysed their transcriptomes using Affymetrix microarrays (Figure 1A–B, Figure 1—figure supplement 1). At this stage of development, it is not possible to predict the polarity of the embryo with complete certainty. To prevent contamination of the samples, we designed a verification strategy by which the predicted posterior and anterior explants were collected, the rest of the embryo immediately fixed and then processed for in situ hybridisation for *cVg1*, developing the colour reaction for long enough to detect residual *cVg1* expression around the posterior explant site. From each set of 40 embryos, approximately 36 had been dissected correctly; the explants from the remainder (3 × 4) were discarded (Figure 1—figure supplement 1). Each set of verified PMZs and AMZs (3 × 36 of each) was then pooled and run on Affymetrix 30K chicken microarrays.

**Figure 1—figure supplement 1.**
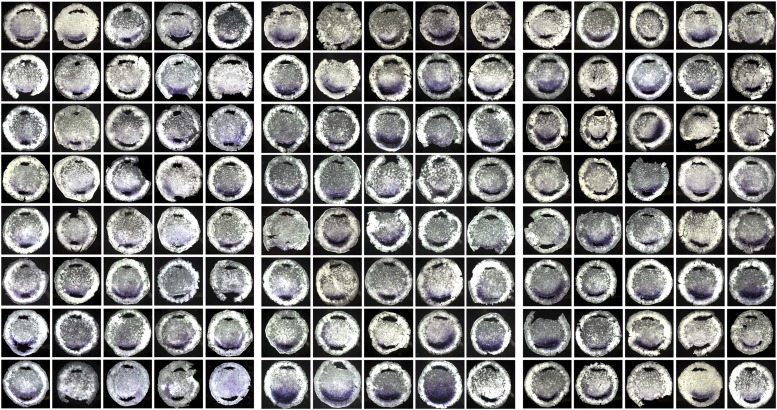
The 120 embryos used for the first screen (AMZ vs PMZ), after excision of the explants and ISH for *cVg1*. The three sets of 40 embryos correspond to those used for each Affymetrix microarray. **DOI:** http://dx.doi.org/10.7554/eLife.03743.004

**Figure 1—figure supplement 2.**
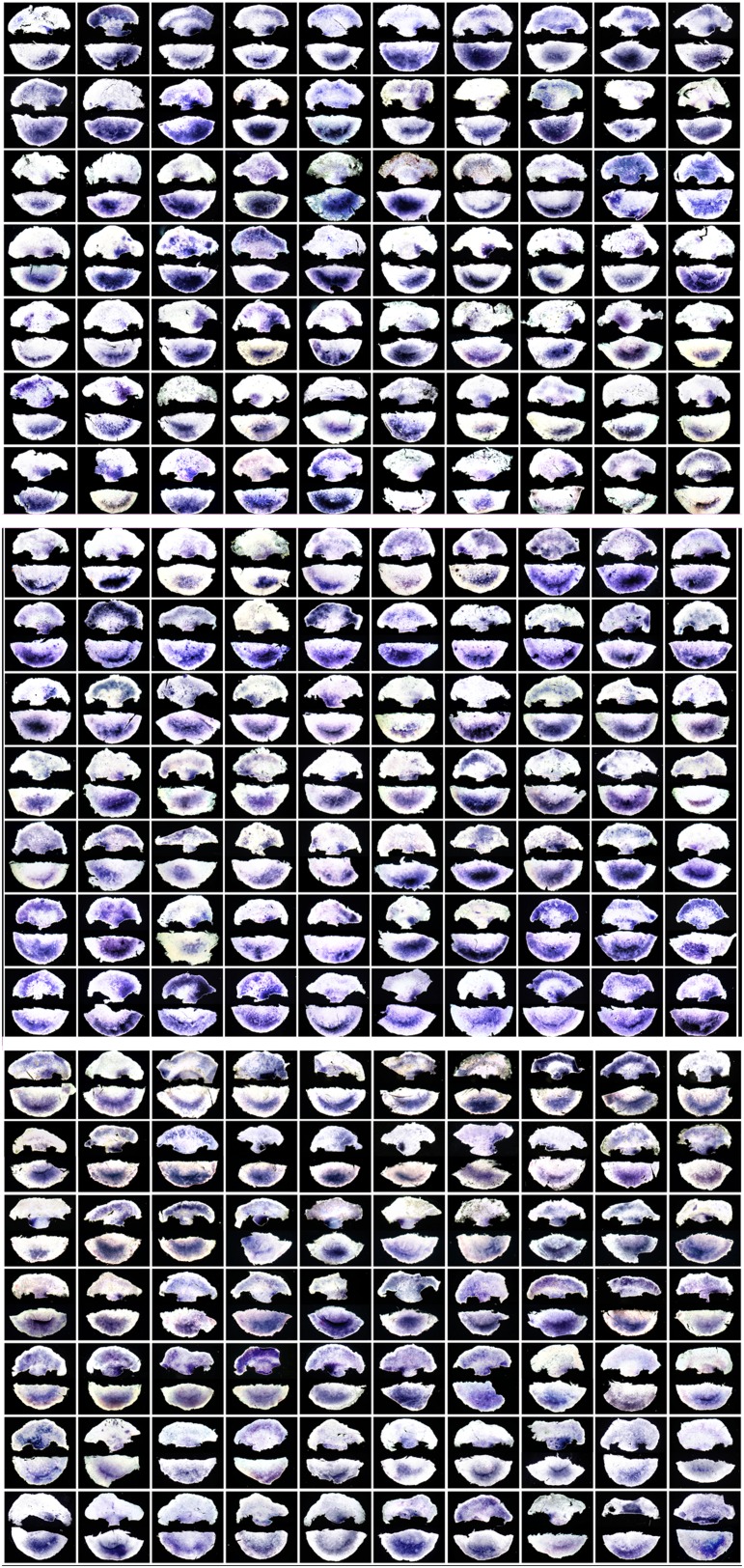
The 210 embryos used for the second screen (lateral marginal zone from isolated anterior half embryos, cultured for 7 hr, then sorted into *cVg1*-like or *cVg1*-unlike expression). The procedure shown in Figure 1C was used. Embryos (stage XI–XII) were bisected at right angles to the midline, the posterior half (shown at the bottom of each panel) fixed immediately and processed by ISH for *cVg1* to confirm the orientation of the cut. The anterior half was cultured for 7 hr, a piece of marginal zone adjacent to the cut edge excised from each side, and the remainder of the anterior half fixed and processed for *cVg1* expression (upper image in each panel). The pieces corresponding to the side expressing *cVg1* were pooled together (‘*cVg1*-like’), and those on the opposite side pooled with each other (‘cVg1-unlike’). Seventy embryos were used for each microarray, and the experiment performed in triplicate. The sets of embryo fragments shown here correspond to each of the three microarrays. **DOI:** http://dx.doi.org/10.7554/eLife.03743.005

**Figure 1—figure supplement 3.**
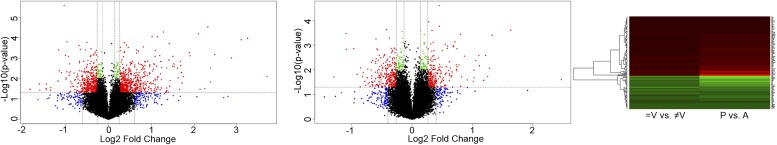
Analysis of microarray datasets for posterior marginal zone (PMZ) and isolated anterior cut halves. Volcano plots showing the correlation between p values and fold change of probes for each microarray experiment. The left graphs displays gene expression data from whole embryos (PMZ vs AMZ), and the second graph displays data from the anterior cut halves (*cVg1*-like and -unlike). Probes that pass a p value threshold of 0.05 together with a fold change cut-off of 1.2 (0.263 log_2_(FC)) are coloured red. Those that do not pass the p value threshold of 0.05 but pass the fold change cut-off of 1.2 (0.263 log_2_(FC)) are blue. Those that pass the p value threshold of 0.05 but not the fold change cut-off of 1.2 (0.263 log_2_(FC)) are green. The heat map on the right side of the figure uses hierarchical clustering to compare the 122 upregulated (red) and 78 downregulated (green) probes from the PMZ (P vs A) and the isolated anterior cut halves expressing *cVg1* (=V vs ≠V). **DOI:** http://dx.doi.org/10.7554/eLife.03743.006

Next, we used a similar strategy for isolated anterior halves of embryos. It was previously reported that *cVg1* starts to be expressed in either the left or right corner of the cut anterior half around 6 hr after bisection (Bertocchini et al., 2004). We chose to collect the left and right corners of the marginal zone adjacent to the cut edge 7 hr following bisection to ensure that surrounding *cVg1* expression could be detected after excision of the fragment. To confirm that all embryos had been cut at exactly right angles to the future axis, the posterior half of each embryo was fixed immediately after cutting and subjected to in situ hybridisation for *cVg1*. The anterior half was cultured for 7 hr, the left and right corners of the margin were dissected, and the remainder was fixed and processed for *cVg1* expression (with extended reaction time to detect weak expression). We estimated that 70 explants would be needed for microarray analysis of each sample; this was done in triplicate (Figure 1C–D; Figure 1—figure supplement 2). Samples were designated ‘*cVg1*-like’ or ‘*cVg1*-unlike’ based on this and pooled accordingly. This strategy randomised any left–right asymmetric genes unrelated to axial polarity and regulation, and enriched those for the cells in which *cVg1* was just starting to be expressed de novo in one sample, and their contralateral equivalents (not expressing *cVg1*) in the other. RNA from the pooled explants (approximately 3 × 63 of each type) was analysed using Affymetrix chicken microarrays.

The intersection between the two datasets from the above screens was used to identify genes co-regulated with *cVg1*, as well as those that are enriched in equivalent regions *not* expressing *cVg1*, both in normal embryos and during regulation (Figure 1B,D,E and Figure 1—figure supplement 3). Using a threshold of just 1.2-fold change and p < 0.05, this strategy identified 122 sequences (corresponding to 85 genes) with putative ‘cVg1-like’ expression (a c*Vg1*-synexpression group) and 78 sequences (52 genes) expressed more highly in the ‘Vg1-unlike’ explants (cVg1 negative) . A list of the top common genes ranked by fold change is shown in Table 1. Comparison of the top ‘Vg1-like’ candidates from whole embryos (PMZ vs AMZ) with their counterparts from half-embryos shows highly significant correlation (Spearman's rank Rho = 0.73; p = 0.00036). Confirming that the screen was performed appropriately, *cVg1* (incorrectly annotated as *GDF3* instead of *GDF1* in the current version of the chicken genome, Galgal4) itself appears among the top genes: it is upregulated 4.3 fold, with a p value of 0.00006 (rank 11) in whole embryos, and 1.71, p = 0.0062 in the isolated anterior half (rank 21). Among all genes, *Pitx2* immediately stands out as the best candidate, being very strongly co-regulated with *cVg1* and the top transcription factor on the list. In the PMZ of whole embryos *Pitx2* is upregulated almost 10-fold compared to the AMZ explants (three different probes, ranking two, three and eight on the list; p = 0.0001, 0.00019 and 0.003 respectively), whereas in the cut halves it is upregulated by about 2.4 fold (three probes ranking four, six and eight on the list; p between 0.002–0.008).

**Table 1.** Genes identified from the screens, sorted according to their ranking in PMZ>AMZ in whole embryos **DOI:** http://dx.doi.org/10.7554/eLife.03743.007

		Posterior vs anterior			Anterior half, Vg1-like		
Gene symbol	Probe ID	Fold change	p Value	Rank	Fold change	p Value	Rank
**ADMP**	Gga.354.1.S1_at	13.095	0.0080	1	3.771	0.0686	2
**PITX2**	Gga.3398.2.S1_a_at	9.586	0.0001	2	2.162	0.0069	8
**PITX2**	Gga.3398.1.S1_a_at	8.528	0.0001	3	2.429	0.0087	4
**THPO**	GgaAffx.21801.1.S1_at	7.398	0.0009	4	2.272	0.0267	7
**ST6-GAL2**	Gga.14379.1.S1_at	6.896	0.0744	5	2.506	0.0003	3
**T**	Gga.3772.1.S1_a_at	6.388	0.0867	6	5.544	0.0251	1
**PKDCC**	Gga.12157.1.S1_at	5.618	0.0006	7	1.572	0.2048	33
**PITX2**	Gga.3398.1.S1_at	5.263	0.0030	8	2.320	0.0026	6
**MIXL1**	Gga.426.1.S1_s_at	5.197	0.0160	9	2.378	0.0185	5
**n/a**	Gga.2705.1.S1_at	5.019	0.00002	10	1.415	0.0237	63
**GDF3**	Gga.4324.2.S1_a_at	4.309	0.00006	11	1.710	0.0062	21
**PMEPA1**	Gga.6268.1.S1_at	4.141	0.0805	12	2.032	0.0103	12
**PMEPA1**	GgaAffx.12721.1.S1_at	3.777	0.0007	13	1.745	0.0003	19
**TBX6**	Gga.466.1.S1_at	3.694	0.0004	14	2.030	0.0210	13
**FGF8**	Gga.661.1.S1_at	3.277	0.0052	15	1.690	0.0859	22
**Ovoinhibitor**	Gga.6976.1.S1_at	3.213	0.0015	16	1.467	0.0633	50
**ELK3**	Gga.4498.1.S1_s_at	3.094	0.0096	17	1.503	0.0010	43
**LITAF**	Gga.3383.1.S2_at	3.066	0.0031	18	1.670	0.0110	25
**LITAF**	Gga.3383.1.S1_at	2.895	0.0019	19	1.822	0.0027	17
**n/a**	Gga.13092.1.S1_at	2.823	0.0025	20	1.406	0.0284	66

List of the top 20 common upregulated probes expressed in both the PMZ of whole embryo and isolated anterior cut halves. Entries in red are probes that pass a fold change cut-off of 1.2 as well as a p value cut-off of 0.05; those in blue pass the fold change cut off of 1.2 but not the p value cut-off of 0.05; and those in black pass the p value cut-off but not the fold change. Common genes are ranked according to the fold change of genes expressed in the PMZ (Spearman's rank Rho = 0.72, p = 0.00048).

**Figure 2.**
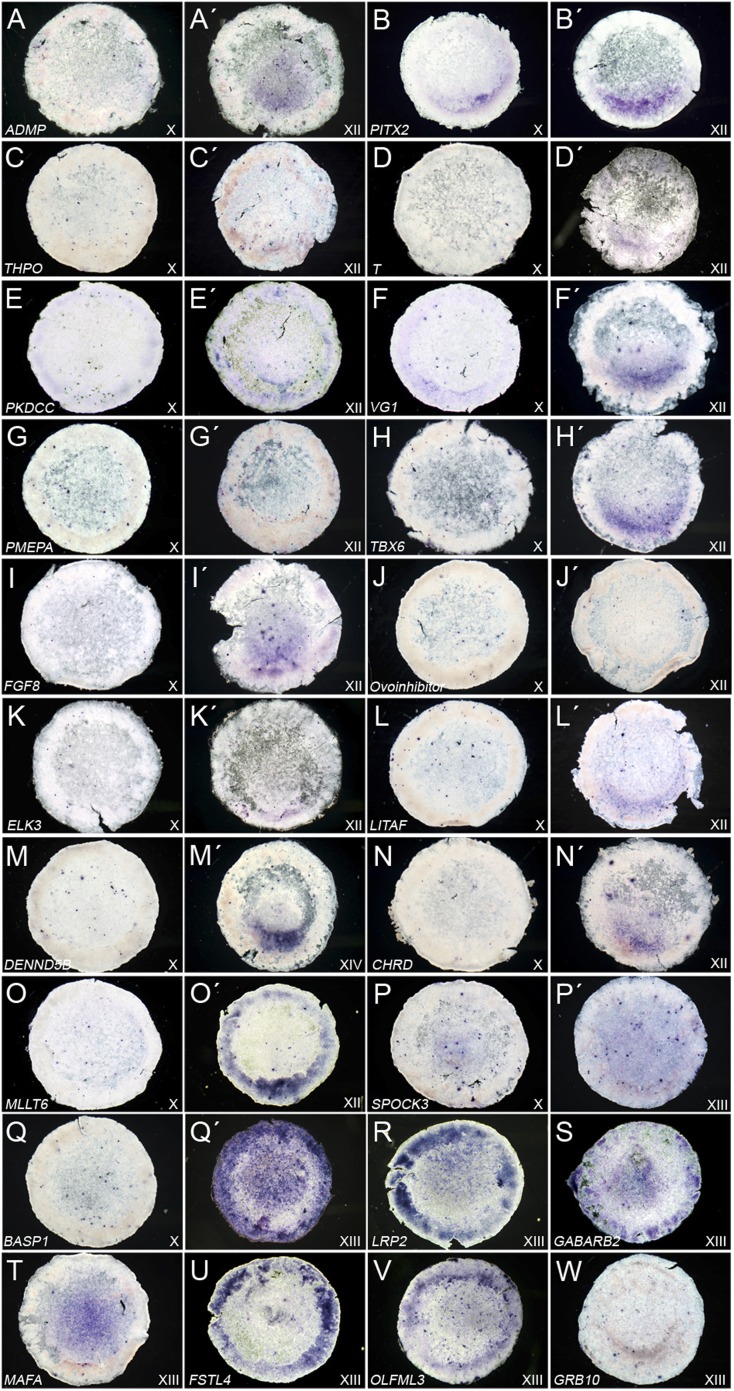
Expression of ‘*cVg1*-like’ and ‘*cVg1*-unlike’ genes, verified by in situ hybridisation. Embryos at stage X–XIII (the earliest stage at which differential expression was detected is shown) were processed using in situ hybridisation for genes co-regulated with *cVg1* (‘*cVg1*-like’, Table 1A–B (**A**–**O**) and genes expressed at lower level in the *cVg1*-positive region than in its counterpart (‘*cVg1*-unlike’, Table 1C–D) (**P**–**W**), from the two microarray screens (see Table 1C–D). The expression of 23 genes (15 ‘*cVg1*-like’ and 8 ‘*cVg1*-unlike’) is shown here. **DOI:** http://dx.doi.org/10.7554/eLife.03743.008

To confirm the microarray results, we examined 53 of the differentially expressed genes by whole-mount in situ hybridisation at pre-primitive streak stages X-XIII (Eyal-Giladi and Kochav, 1976) (23 of these are shown in Figure 2). Apart from *Pitx2* three other genes show a similar expression to *cVg1* at stage XII: *Elk3* (an Ets-domain protein also known as SRF accessory protein-2), *PKDCC* (protein kinase domain containing cytoplasmic protein) and *LITAF* (lipopolysaccharide-induced tumor necrosis factor-alpha). Others are expressed in cells adjacent to the PMZ (and are therefore likely to represent early axial cells), such as *ADMP*, *Brachyury* (*T*), *Mixl1*, *Tbx6*, *FGF8,* and *CHRD*, or are expressed much later (stage XIV), such as *DENND5B*. A final group is virtually undetectable, such as *Thrombopoietin*, *Ovoinhibitor* and *PMEPA*. All of these rank lower than *Pitx2* (see above and Table 1, Table2, Table3, Table 4): *Elk3* ranks 17^th^ in whole embryos and 43^rd^ in cut halves, *PKDCC* ranks 7^th^ in whole embryos and 33^rd^ in the anterior half, and *LITAF* ranks 18–19^th^ in whole embryos and 17^th^ and 25^th^ in anterior halves. *Pitx2* is therefore the strongest candidate as a putative regulator of *cVg1*. The ‘*cVg1*-unlike’ genes (Figure 2P–W) give less obvious information. Comparison of the top genes identified from differential expression in whole embryos (stronger in AMZ than in PMZ) with their counterparts in the corners of anterior halves reveals weak correlation (Spearman's rank Rho between −0.02 and 0.17; p = 0.44–0.93). These genes include those encoding extracellular matrix proteins as well as glucose-, glutamate-, glycine-, GABA- and LDL transporters and receptors, the transcriptional repressor ID3, and BASP1, which has been reported to act as a transcriptional co-suppressor for WT1 (Carpenter et al., 2004), among others. In situ hybridisation for these genes does not show enrichment in the AMZ or any other obvious pattern consistent with a putative role as an inhibitor of c*Vg1* expression in the PMZ at the appropriate stages of development (Figure 2P–W). *Pitx2* therefore remains as the most likely candidate.

**Table 2.** Genes identified from the screens, sorted according to their ranking in *cVg1*-like > -unlike in isolated anterior halves **DOI:** http://dx.doi.org/10.7554/eLife.03743.009

		Anterior half, Vg1-like			Posterior vs anterior		
Gene symbol	Probe ID	Fold change	p Value	Rank	Fold change	p Value	Rank
**T**	Gga.3772.1.S1_a_at	5.544	0.0251	1	6.388	0.0867	6
**ADMP**	Gga.354.1.S1_at	3.771	0.0686	2	13.095	0.0080	1
**ST6GAL2**	Gga.14379.1.S1_at	2.506	0.0003	3	6.896	0.0744	5
**PITX2**	Gga.3398.1.S1_a_at	2.429	0.0087	4	8.528	0.0001	3
**MIXL1**	Gga.426.1.S1_s_at	2.378	0.0185	5	5.197	0.0160	9
**PITX2**	Gga.3398.1.S1_at	2.320	0.0026	6	5.263	0.0030	8
**THPO**	GgaAffx.21801.1.S1_at	2.272	0.0267	7	7.398	0.0009	4
**PITX2**	Gga.3398.2.S1_a_at	2.162	0.0069	8	9.586	0.0001	2
**DENND5B**	Gga.16679.1.S1_at	2.151	0.0026	9	2.341	0.1952	27
**WNT8A**	Gga.886.1.S1_at	2.078	0.0042	10	1.931	0.0259	53
**CHRD**	Gga.490.1.S1_at	2.064	0.0739	11	2.267	0.0959	30
**PMEPA1**	Gga.6268.1.S1_at	2.032	0.0103	12	4.141	0.0805	12
**TBX6**	Gga.466.1.S1_at	2.030	0.0218	13	3.694	0.0004	14
**HOXB1**	Gga.18352.1.S1_at	2.015	0.0298	14	1.749	0.0029	73
**AREGB**	GgaAffx.6867.1.S1_at	1.891	0.0526	15	1.640	0.1195	85
**SOHO-1**	Gga.770.1.S1_at	1.884	0.0256	16	1.417	0.1282	106
**LITAF**	Gga.3383.1.S1_at	1.822	0.0027	17	2.895	0.0019	19
**MLLT6**	Gga.11449.1.S1_at	1.762	0.0194	18	2.218	0.0091	32
**PMEPA1**	GgaAffx.12721.1.S1_at	1.745	0.0003	19	3.777	0.0007	13
**FAM19A1**	Gga.11944.2.S1_a_at	1.737	0.0948	20	2.102	0.0956	41

List of the top 20 common upregulated probes expressed in both the PMZ of whole embryo and isolated anterior cut halves. Entries in red are probes that pass a fold change cut-off of 1.2 as well as a p value cut-off of 0.05; those in blue pass the fold change cut off of 1.2 but not the p value cut-off of 0.05; and those in black pass the p value cut-off but not the fold change. Common genes are ranked according to the fold change of genes in the anterior cut halves (Spearman's rank Rho = 0.73, p = 0.00036).

**Table 3.** Genes identified from the screens, sorted according to their ranking in AMZ > PMZ in whole embryos **DOI:** http://dx.doi.org/10.7554/eLife.03743.010

		Posterior vs anterior			Anterior half, Vg1-like		
Gene symbol	Probe ID	Fold change	p Value	Rank	Fold change	p Value	Rank
**LPL**	Gga.4248.1.S1_at	−3.578	0.0350	1	−1.217	0.0191	69
**UPK1B**	Gga.17532.1.S1_at	−2.573	0.0662	2	−1.519	0.0046	15
**UPK1B**	Gga.17532.1.S1_s_at	−2.550	0.0383	3	−2.133	0.0003	2
**SPOCK3**	GgaAffx.6009.1.S1_at	−2.531	0.0284	4	−1.461	0.0174	24
**SLC1A3**	GgaAffx.25896.1.S1_at	−2.333	0.2168	5	−1.338	0.0272	47
**SLC5A1**	Gga.8594.1.S1_at	−2.284	0.2814	6	−2.253	0.0857	1
**BASP1**	Gga.3179.1.S1_at	−2.276	0.0916	7	**−1.170**	**0.0079**	75
**ID3**	Gga.4048.1.S1_at	−2.243	0.0004	8	−1.422	0.0463	34
**ATP13A4**	GgaAffx.12270.1.S1_at	−2.172	0.0486	9	−1.202	0.0148	71
**DIO3**	Gga.552.1.S1_at	−2.149	0.0051	10	−1.656	0.0376	12
**UPK1B**	Gga.12930.1.S1_at	−2.134	0.0640	11	−2.123	0.0014	3
**COL4A2**	Gga.3104.1.S1_at	−2.115	0.1024	12	−1.286	0.0059	58
**SLC1A3**	GgaAffx.25896.1.S1_s_at	−2.089	0.3192	13	−1.241	0.0363	60
**LRP2**	GgaAffx.23355.1.S1_at	−2.052	0.0637	14	−1.306	0.0144	55
**EFNB2**	Gga.13001.1.S1_s_at	−2.049	2.6527	15	−1.360	0.0220	42
**---**	Gga.15960.1.S1_at	−2.005	0.0007	16	−1.437	0.0203	29
**---**	Gga.18649.1.A1_at	−1.991	0.0092	17	−1.358	0.0068	43
**GABRA1**	Gga.17167.1.S1_at	−1.955	0.1060	18	−1.487	0.0978	20
**GLRA3**	GgaAffx.6806.1.S1_at	−1.951	0.0001	19	−1.672	0.0209	10
**DIO2**	Gga.1819.1.S1_at	−1.908	0.1217	20	−1.457	0.0025	25

List of the top 20 downregulated probes common to both the PMZ of whole embryo and isolated anterior cut halves. Entries in red are probes that pass a fold change cut-off of −1.2 as well as a p value cut-off of 0.05; those in blue pass the fold change cut off of −1.2 but not the p value cut-off of 0.05; and those in black pass the p value cut-off but not the fold change cut-off. Common genes are ranked according to the fold change of genes in the PMZ (Spearman's rank Rho = −0.02, p = 0.93).

**Table 4.** Genes identified from the screens, sorted according to their ranking in Vg1-unlike > Vg1-like in isolated anterior halves **DOI:** http://dx.doi.org/10.7554/eLife.03743.011

		Anterior half, Vg1-like			Posterior vs anterior		
Gene symbol	Probe ID	Fold change	p Value	Rank	Fold change	p Value	Rank
**SLC5A1**	Gga.8594.1.S1_at	−2.253	0.0857	1	−2.284	0.2814	6
**UPK1B**	Gga.17532.1.S1_s_at	−2.133	0.0003	2	−2.554	0.0383	3
**UPK1B**	Gga.12930.1.S1_at	−2.123	0.0014	3	−2.134	0.0642	11
**EDNRB**	Gga.3306.1.S1_s_at	−1.935	0.1491	4	−1.773	0.2348	32
**FSTL4**	Gga.13574.1.S1_at	−1.878	0.0152	5	−1.496	0.0014	51
**GABARB2**	Gga.17131.1.S1_at	−1.814	0.0491	6	−1.586	0.0647	45
**KCNAB1**	Gga.4971.1.S1_at	−1.763	0.0185	7	−1.302	0.0238	72
**MYLK**	Gga.6776.1.S1_at	−1.758	0.0126	8	−1.822	0.1777	30
**CBLN4**	GgaAffx.4848.1.S1_s_at	−1.702	0.0424	9	−1.641	0.0564	40
**GLRA3**	GgaAffx.6806.1.S1_at	−1.672	0.0209	10	−1.951	0.0001	19
**SLC14A2**	Gga.7955.1.S1_at	−1.663	0.0094	11	−1.897	0.0561	22
**DIO3**	Gga.552.1.S1_at	−1.656	0.0376	12	−2.149	0.0051	10
**KCNMA1**	Gga.19342.1.S1_at	−1.599	0.2068	13	−1.384	0.0097	63
**GRB10**	GgaAffx.8324.2.S1_at	−1.534	0.0569	14	−1.213	0.0236	76
**UPK1B**	Gga.17532.1.S1_at	−1.519	0.0046	15	−2.573	0.0662	2
**CALD1**	GgaAffx.21386.1.S1_s_at	−1.518	0.0054	16	−1.727	0.1106	33
**MAFA**	Gga.974.1.S1_at	−1.518	0.2597	17	−1.855	0.0144	29
**OLFML3**	Gga.1150.2.S1_a_at	−1.509	0.0035	18	−1.382	0.0149	62
**---**	Gga.18986.1.S1_at	−1.487	0.0109	19	−1.522	0.0312	48
**GABRA1**	Gga.17167.1.S1_at	−1.487	0.0978	20	−1.955	0.1067	18

List of the top 20 downregulated probes common to both the PMZ of whole embryo and isolated anterior cut halves. Entries in red are probes that pass a fold change cut-off of −1.2 as well as a p value cut-off of 0.05; those in blue pass the fold change cut off of −1.2 but not the p value cut-off of 0.05; and those in black pass the p value cut-off but not the fold change cut-off. Common genes are ranked according to the fold change of genes in the anterior cut halves (Spearman's rank Rho = 0.17, p = 0.44).

**Figure 3.**
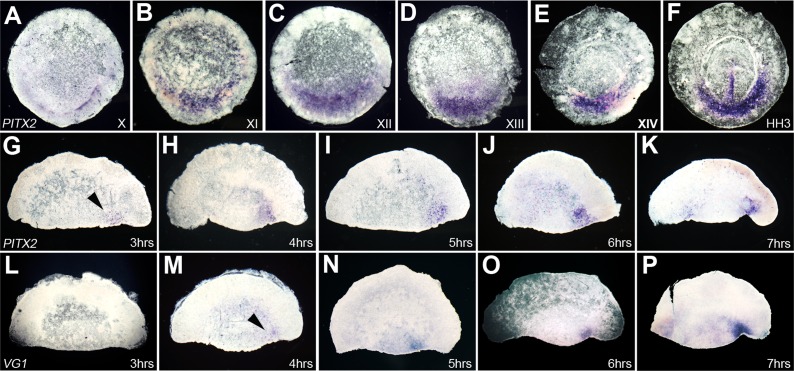
Time-course of expression of *Pitx2* in whole embryos and in isolated anterior halves, and comparison with *cVg1*. (**A**–**F**) Time-course of *Pitx2* expression in whole embryos. Expression is restricted to the posterior marginal zone (PMZ) already at the time of laying (stage X); this intensifies over the subsequent stages. At primitive streak stages (HH2–3), expression is seen in the primitive streak itself as well as in the PMZ (F). (**G**–**P**) Comparison of the time-course of *Pitx2* (**G**–**K**) and *cVg1* (**L**–**P**) upregulation in isolated anterior halves. *cVg1* is first detectable 4–5 hr after cutting (**M**), whereas *Pitx2* can be detected an hour earlier (**G**). Both *Pitx2* and *cVg1* expression appears randomly on the left or the right corner of the marginal zone adjacent to the cut edge. The series chosen for this figure shows upregulation on the right side of all embryo fragments. **DOI:** http://dx.doi.org/10.7554/eLife.03743.012

To determine the temporal relationship between *Pitx2* and *cVg1* in whole embryos and during embryonic regulation, we compared their expression in time-course. In normal embryos *cVg1* is first detected at around stage XI (Bertocchini and Stern, 2012). We detected *Pitx2* in the PMZ by the time of laying, stage X (Figure 3A), where it remains until early streak stages (Figure 3B–F). In isolated anterior halves, we increased the sensitivity of the assay by developing the NBT/BCIP colour reaction for several days to ensure that even weak expression could be detected. With this strategy we detected *cVg1* expression 4–5 hr after cutting (Figure 3L–P), 1–2 hr earlier than in previous reports (Bertocchini et al., 2004), while *Pitx2* appeared even earlier, just 3 hr after embryo bisection (Figure 3G–K). Taken together, these results implicate *Pitx2* as a good candidate for an upstream regulator of *cVg1* expression: it is a transcription factor, it is expressed in the same domain as *cVg1* in whole embryos and in bisected embryo marginal zone, and it is expressed before *cVg1*.

### Pitx2 is required for axis development and embryonic regulation

**Figure 4.**
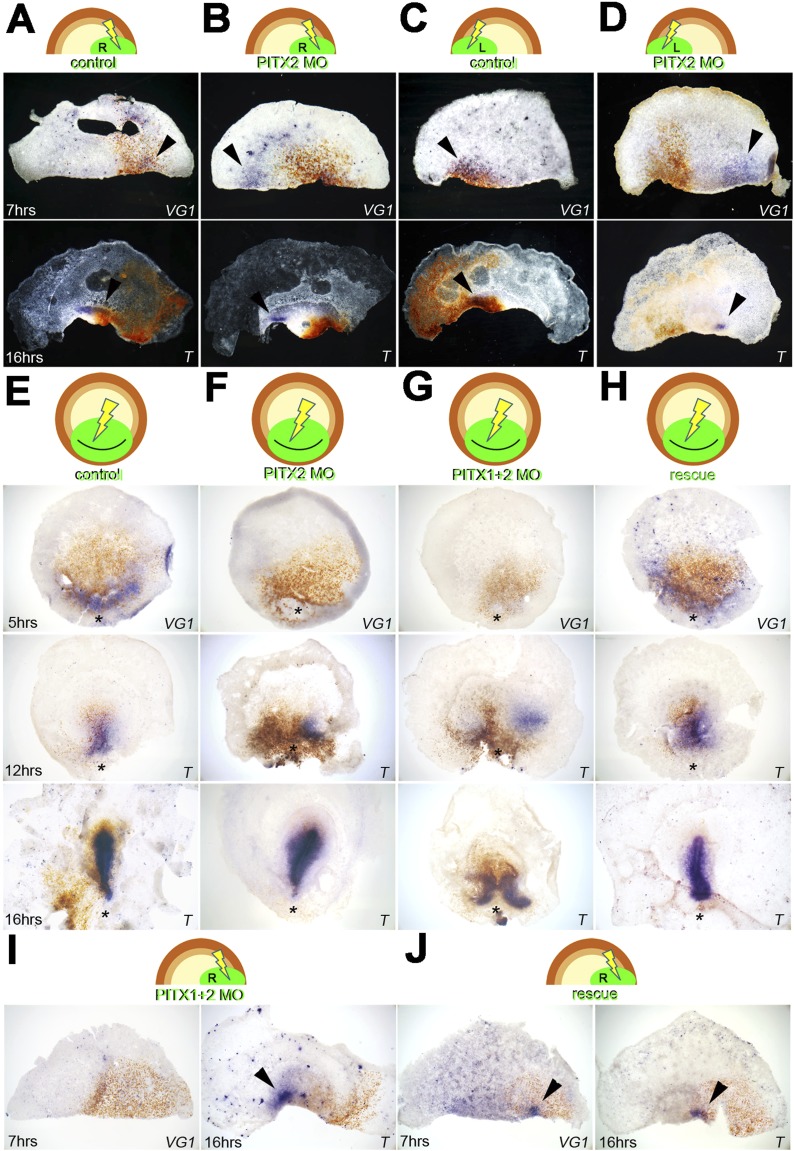
Pitx2 is required for *cVg1* expression and for axis formation. (**A**–**D**) A morpholino (MO) targeting Pitx2 applied to one side of the marginal zone of an isolated anterior half shifts axis formation to the opposite marginal zone. (**A–B**) The experiment done on the right side (A = control-MO, B=Pitx2-MO), (**C**–**D**) the equivalent done on the left marginal zone. The top row shows diagrams of the experiment, the middle row shows embryo fragments 7 hr after electroporation/cutting, hybridised for *cVg1* (purple) and stained with anti-fluorescein (in the MO, brown), and the lower row shows fragments processed for *Brachyury* (*T*) in purple and fluorescein in brown. (**E**–**F**) In whole embryos, *Pitx2*-MO has a transient effect. At 5 hr, *cVg1* expression is lost, at 12 hr a primitive streak (*T*-expressing) is sometimes seen especially at the edge of the electroporated domain, but by 16 hr embryos appear essentially normal. (**E**) Shows embryos electroporated with control-MO, F are embryos transfected with Pitx2-MO. (**G–H**). Embryos electroporated with MOs targeting both Pitx2 and Pitx1 do not recover: at 5 hr, no *cVg1* is seen; at 12–16 hr there is a high proportion of embryos with either no streak or two *T*-expressing streaks arising from outside the MO-electroporated domain (as shown in **G**). This effect can be rescued by co-electroporation of Pitx2 with the mixture of *Pitx1*-MO/*Pitx2*-MO (**H**). (**I**–**J**) Likewise, in isolated anterior half-embryos, the effects of electroporation of the *Pitx1*-MO/*Pitx2*-MO combination (**I**) can be rescued by co-electroporation with *Pitx2* alone (**J**): *cVg1* expression is now seen on the electroporated side. Arrowheads point to sites of expression. **DOI:** http://dx.doi.org/10.7554/eLife.03743.013 10.7554/eLife.03743.014Figure 4—source data 1.Numbers of embryos displaying different types of results in the Pitx loss-of-function experiments illustrated in the main Figure.Each type of experiment is summarised in a separate sub-table, where each row refers to a particular column (**A**–**J**) and row (first, second, and third) of panels in Figure 4. The table columns show each type of result obtained: ‘Vg1 R’ refers to embryos showing *cVg1* expression on the right side, ‘streak L’ when there is a *Brachyury*-expressing primitive streak on the left, ‘streak L/R’ when there are two streaks (one arising from each side), etc.**DOI:** http://dx.doi.org/10.7554/eLife.03743.014

To determine whether Pitx2 is important for embryonic regulation and for controlling *cVg1* expression, we used targeted electroporation of morpholino oligonucleotides (MOs). When a translation-blocking *Pitx2*-MO was targeted to the right edge of an isolated anterior half embryo, the frequency of axis formation shifted to the opposite side (Figure 4A–D; Figure 4—source data 1A,B): embryos electroporated with control-MO at the right edge had random *cVg1* expression on the right, left or neither edges in equal proportion. In contrast, *Pitx2*-MO on the right generated a majority (9/10, p = 0.05) of embryos with no expression and the remaining embryo with expression on the opposite side 7 hr post-electroporation (Figure 4B, Figure 4—source data1B). After 16 hr (Figure 4—source data1B) embryos electroporated with control-MO at the right edge formed a primitive streak expressing *Brachyury* randomly; with *Pitx2*-MO on the right (Figures 4B), 8/10 embryos formed a streak on the left side and 2 had two streaks (one from each corner), but no embryo formed a single streak arising from the MO-transfected side (hypergeometric exact test with 2 × 3 contingency table, p = 0.026). Equivalent results were observed with MO electroporations on the left (Figure 4C–D; Figure 4—source data1A,B): control-MO embryos transfected on the left and examined for *cVg1* expression had random expression on the right, whereas for *Pitx2*-MO *cVg1* expression was biased towards the left. After 16 hr, control embryos transfected on the left formed a *Bra*-expressing streak on the right, again randomly, while *Pitx2-*MO embryos formed a streak on the right in 2/8 cases, none on the left, 1/8 on both sides and 5/8 with no streak (p = 0.003) (Figure 4D, Figure 4—source data1B).

**Figure 4—figure supplement 1.**
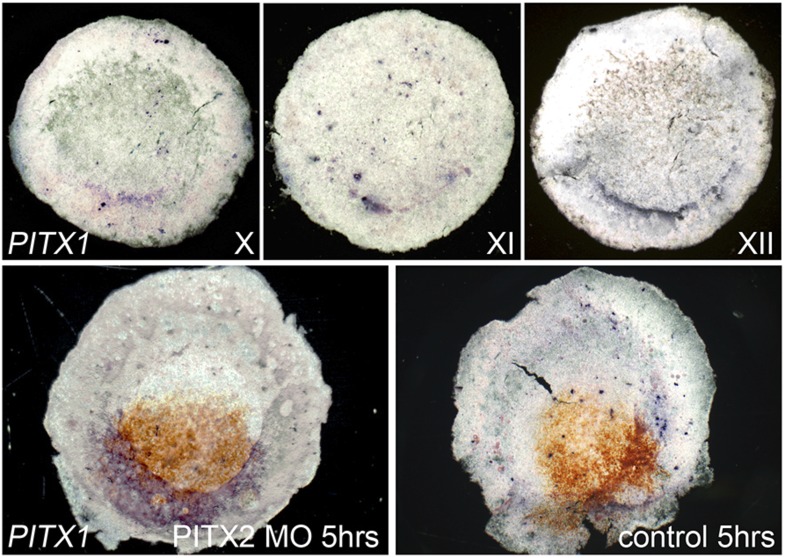
*Pitx1* is expressed posteriorly in normal embryos, and upregulated in Pitx2 morphants. The upper row of images show embryos at stages X, XI and XII processed for in situ hybridisation for *Pitx1*. The left lower embryo shows upregulation of *Pitx1* (blue), 5 hr after electroporation of *Pitx2*-MO (brown from anti-fluorescein staining). The right lower image shows lack of effect of similar electroporation of a control-MO. **DOI:** http://dx.doi.org/10.7554/eLife.03743.015

In intact embryos electroporated at stage X, *Pitx2*-MO affected *cVg1* expression and streak formation (Figure 4E–F; Figure 4—source data 1C–D). Control-MO targeted to the PMZ did not alter *cVg1* expression after 5 hr or streak formation after 12–16 hr (Figure 4E; Figure 4—source data1C,D). With *Pitx2*-MO, *cVg1* expression was affected after 5 hr (0/6 embryos expressing; p < 0.001). Embryos started to recover, however, at later time points: at 12 hr, 3/12 had a normal streak, 3/12 had a displaced streak, 3/12 had two streaks, and the remaining 3/12 had no streaks (p = 0.1—not significantly different). By 16 hr, the majority of the embryos were normal (7/8; the remaining embryo had a displaced streak) (Figure 4F; Figure 4—source data 1D). These results suggest that while knockdown of *Pitx2* affects *cVg1* expression, embryos tend to recover by 12–16 hr. We reasoned that functional redundancy with another *Pitx* gene, or compensatory upregulation of such a gene in response to *Pitx2* knockdown, could account for this recovery. To test this, we examined *Pitx1* expression in normal embryos at stages X–XII and in embryos electroporated with *Pitx2*-MO. *Pitx1* is barely detectable in the PMZ at stage X–XII (Figure 4—source data 1). After *Pitx2*-MO electroporation, expression increased considerably (6/6 embryos; Figure 4—source data 1). We therefore repeated the targeting experiments in whole embryos using a mixture of two MOs targeting the translation start site of *Pitx2* and an internal splice junction of *Pitx1*, respectively. *Pitx1*+*2*-MOs caused loss of *cVg1* at 5 hr in 6/6 cases (p = 0.005). By 12 hr 1/9 embryos had a normal streak, 3/9 had a displaced streak, 3/9 had two streaks and 2/9 had none (p = 0.043). By 16 hr no recovery was observed: 4/4 embryos had double streaks, neither arising from the targeted site (p = 0.029; Figure 4G; Figure 4—source data 1E,F). This effect could be rescued by supplying *Pitx2* alone: co-electroporation of *Pitx1*+*2*-MO together with a *Pitx2* expression construct lacking the MO target sequence led to normal axis formation: in 5/6 cases *cVg1* expression was restored after 5 hr incubation and by 16 hr 12/16 embryos displayed a normal streak (Figure 4H; Figure 4—source data 1G–H). Likewise in isolated anterior halves, the effects of *Pitx1+2*-MO (Figure 4I; Figure 4—source data 1I,J) could be rescued by *Pitx2*. After electroporation on the right, *cVg1* expression was seen on the right in 4/10, on the left in 3/10, and in neither in 3/10 cases (Figure 4J; Figure 4—source data 1K). After 16 hr a primitive streak developed on the right in 3/8 cases, on the left in 2/8 and no streak in 3/8 cases (Figure 4—source data l).

In conclusion, Pitx2 is required for expression of *cVg1* in the PMZ as well as for formation of the normal primitive streak. In isolated anterior halves, Pitx2 is required both for *cVg1* expression and for the later formation of a primitive streak. Knockdown of *Pitx2* is followed by upregulation of the related transcription factor *Pitx1*, which can partly compensate for the loss of *Pitx2*.

### A bioinformatics approach to uncover candidate regulatory regions for cVg1

**Figure 5.**
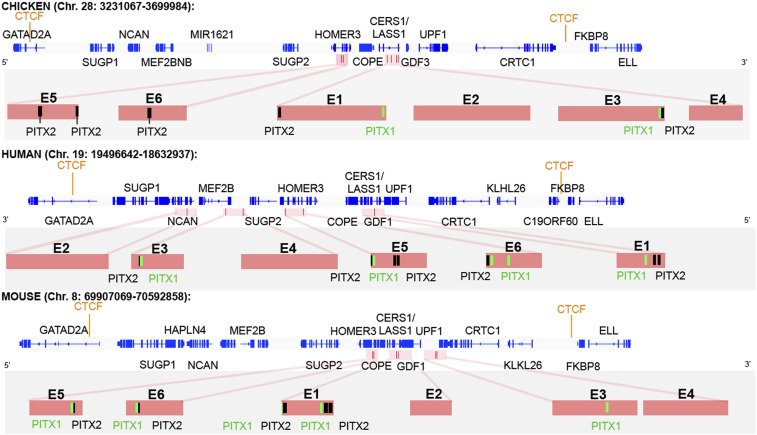
CTCF insulator analysis, enhancer identification and synteny of the *cVg1* locus. The chicken *cVg1* locus with computationally predicted conserved CTCF-binding sites in chick, human, and mouse is shown (genes represented in blue). This putative insulator region lies ∼200 kB upstream and ∼100 kB downstream of *cVg1/GDF3* and harbours other genes such as *CERS1/Lass1*, *COPE*, *DDX49* and *HOMER3* upstream and *UPF1* downstream of *cVg1*. Six putative enhancer regions (E1–E6), predicted using DREiVe, are displayed in pink. In chick, E1 (galGal4 genomic coordinates: *chr28:3502783-3504834*) and E2 (*chr28:3504993-3508041*) lie in the first intron of the bicistronic *CERS1/Lass1* gene. E3 (*chr28:3510154-3511032*) and E4 (chr28:3,511,413-3,511,725) lie in intron 4 of *CERS1/Lass1* and E5 (*chr28:3471200-3471520*) and E6 (*chr28:3,471,946-3,472,230*) respectively lie in introns 1 and 2 of *HOMER3*. E1 and E3 each contain conserved Pitx1 (black) and Pitx2 (green) binding sites. E2 and E4 do not contain any Pitx sites and E5 and E6 contain Pitx2-binding sites but no Pitx1-binding sites. The orthologous regions of human (chromosome 19) and mouse (chromosome 8) genomes are also shown. Note that the corresponding human region has been inverted, and that although all six elements are found within it, these appear in different order and are associated with different introns and intergenic regions than in chick and mouse. **DOI:** http://dx.doi.org/10.7554/eLife.03743.016

The *cVg1* gene (erroneously annotated as *GDF3* in the chick genome; its orthologue is human *GDF1*, as confirmed by synteny; see Figure 5) is located on chicken chromosome 28. As a parallel approach to the above to identify putative upstream regulators, we applied a recently described pipeline (Khan et al., 2013), starting with prediction of conserved, constitutive CTCF-binding sites (CTCF is an 11 zinc-finger transcriptional repressor protein that co-localizes with cohesin and acts to delimit chromatin loops) (Holwerda and de Laat, 2013; Merkenschlager and Odom, 2013) around this locus which could act as insulators. This was followed by algorithms to identify conserved motifs in non-coding regions that are order independent, modified from the Enhancer Discovery using only Genomic Information (EDGI) tool described for Drosophila (Sosinsky et al., 2007).

**Figure 5—figure supplement 1.**
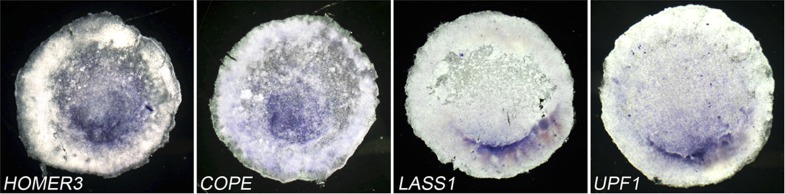
Expression of genes adjacent to *cVg1/GDF3* on chick chromosome 28. Expression of *Lass1/CERS1*, *HOMER3*, *COPE* and *UPF1* is shown. All are expressed posteriorly although the patterns are not quite identical to each other. **DOI:** http://dx.doi.org/10.7554/eLife.03743.017

The insulator-predicting software identifies strongly conserved CTCF-binding sites about 200 kB upstream and about 100 kB downstream of *cVg1*/*GDF3* (Figure 5). The *cVg1*/*GDF3* gene itself is bicistronic, the upstream exons encoding *Lass1*/*CERS1* and the last two exons containing the cVg1 sequence (Wang et al., 2007). Several other genes lie in this region, including *COPE*, *DDX49* and *HOMER3* upstream and *UPF1* downstream. If the predicted CTCF-binding sites are indeed insulators, we would expect these genes to be co-regulated with *cVg1*. To test this, we examined their expression. Strikingly, all genes examined (*CERS1*/*LASS1*, *COPE*, *HOMER3,* and *UPF1*) are expressed in a similar domain of the PMZ as *cVg1* (Figure 5—figure supplement 1).

We then applied the DREiVe tool (Discovery of Regulatory Elements in Vertebrates, the vertebrate version of EDGI) (Sosinsky et al., 2007; Khan et al., 2013), to identify de novo conserved sequence motifs around this region. This identifies six domains (designated E1–E6), ranging in size from 600–3000 bases, located within the introns of *CERS1*/*LASS1* and of the neighbouring *Homer3* in chick (Figure 5). Analysis of these regions using Position Frequency Matrices from JASPAR and TRANSFAC databases together with the algorithms Matrix-Scan (from RSAT) and Clover predicts four of these regions (E1, E3, E5, and E6) to contain one or more putative binding sites for *Pitx2* and/or the related factor *Pitx1* (Figure 5). The power of DREiVe as a tool for discovering regulatory elements is highlighted by the observation that it is able to identify homologous non-coding regions in the human genome, where the syntenic region (on chromosome 19) is not only inverted but also the orthologous elements are found in a different order, within introns of different neighbouring genes (Figure 5). In mouse (chromosome 8) the arrangement is similar to chick (Figure 5).

### Testing candidate regulatory regions

**Figure 6.**
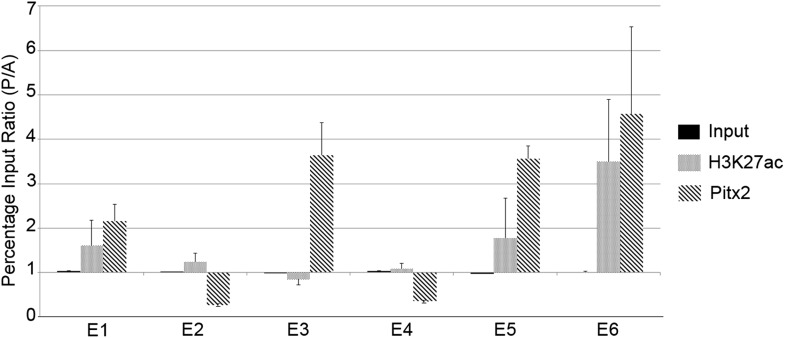
Chromatin immunoprecipitation to test for active histone marks and Pitx2 binding to predicted enhancers. Relative immunoprecipitation around each of the putative six enhancers by an antibody to Pitx2 (diagonal hatching) or an antibody to acetyl-lysine-27 of Histone-H3 (grey shading), expressed as a ratio of the amount precipitated from posterior and anterior marginal zone (PMZ and AMZ) chromatin. Primers were used to target each of the putative enhancers and precipitated chromatin measured by the quantitative polymerase chain reaction (qPCR). Each data bar represents the average of at least three true biological replicates and the error bars indicate standard error of the mean. Amplification from input DNA from each of the same samples is also shown (solid black shading). Note that enhancers that contain Pitx2-binding sites (E1, E3, E5, and E6) are precipitated much more strongly from the PMZ than the AMZ. **DOI:** http://dx.doi.org/10.7554/eLife.03743.018

To determine whether any of the predicted regions bind Pitx2 in the PMZ of normal embryos, we conducted chromatin-immunoprecipitation experiments (ChIP), assessing precipitation by real-time quantitative polymerase chain reaction (qPCR) analysis using primers targeting the predicted regions. We compared chromatin from the AMZ and PMZ (Figure 6). A monoclonal antibody against Pitx2 precipitated chromatin from the PMZ more effectively than from the AMZ for all predicted enhancers that contained consensus Pitx1/2-binding sites (especially E3, E5, and E6) but not those that do not (E2 and E4). These findings suggest that Pitx2 is differentially bound to putative enhancer sites E3, E5, and E6 in the PMZ of normal embryos. We also tested each of the six putative enhancer regions for acetylation of Lys-27 of Histone-3 (H3K27ac), which is associated with active enhancers (Creyghton et al., 2010). Enhancers E5 and E6 showed the greatest differential activity in the PMZ relative to the AMZ. Together, these results suggest that E3, E5, and E6 are the most likely enhancers driving expression in the PMZ.

**Figure 7.**
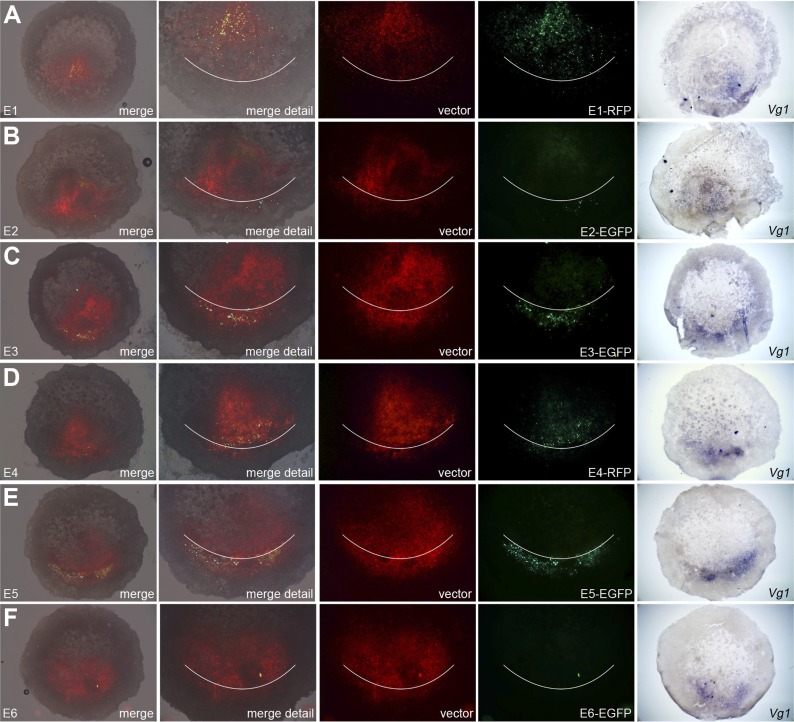
Enhancers E3 and E5 drive expression in the posterior marginal zone of whole embryos. Embryos were electroporated with a construct containing a candidate enhancer (E1–E6), a minimal promoter (TK) and a fluorescent reporter (GFP or RFP), together with a ubiquitous marker (pCAβ-EGFP or DS-RedExpress) to reveal the electroporated area. After 5–9 hr culture the embryos were observed by fluorescence (first 4 columns) and then fixed and processed to reveal *cVg1* expression by in situ hybridisation (last column). The position of Koller's sickle is marked with a curved white line. Enhancers E3 and E5 faithfully recapitulate *cVg1* expression in the posterior marginal zone (PMZ), whereas E1 drives expression inside the embryo (but not in the PMZ) and the remaining enhancers show little or no detectable activity. In all cases, the electroporated area appears red and the activity of the specific enhancer construct in green. **DOI:** http://dx.doi.org/10.7554/eLife.03743.019

**Figure 8.**
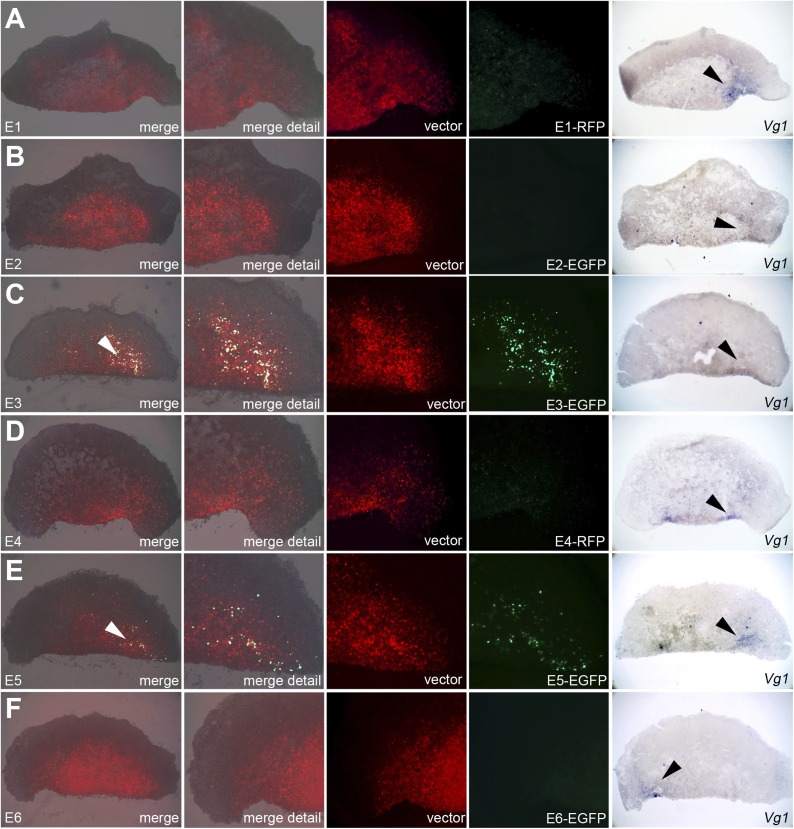
Enhancers E3 and E5 drive expression in the *cVg1*-expressing corner of the marginal zone at the cut edge of isolated anterior half-embryos. Embryos were electroporated with the same vectors as described in Figure 7, then bisected. The anterior half was then cultured for 5–7 hr and viewed under fluorescence (first 4 columns), then fixed and processed for cVg1 expression (last column). Enhancers E3 and E5 drive expression of the reporter at the *cVg1*-expressing edge of isolated anterior half-embryos. Note that unlike what is found in whole embryos, Enhancer E1 does not appear to drive expression in the area pellucida of the isolated anterior half. **DOI:** http://dx.doi.org/10.7554/eLife.03743.021

To test whether these putative enhancers do indeed direct transcription in the correct endogenous domain, we generated reporter constructs based on a vector designed by the group of Kondoh (Uchikawa et al., 2003). Each construct contained one candidate enhancer (E1–E6), a minimal promoter (TK), and a reporter fluorescent protein (EGFP or RFP) and was electroporated either in a very broad domain including the PMZ and lateral marginal zones of normal embryos, or encompassing the entire cut edge (including both corners) of bisected embryos and the anterior half subsequently cultured. A ubiquitous reporter (pCAβ with either EGFP or RFP) was co-electroporated with each construct to reveal the extent of the electroporated domain (Figures 7 and 8). Embryos were then photographed live to reveal the electroporated and expressing domains, then fixed and processed for in situ hybridisation for *cVg1* to determine whether the side with reporter activity corresponds to the *cVg1* expressing region. In whole embryos, E3 and E5 are most efficient in driving expression of the reporter in the PMZ (Figure 7). The same two enhancers also drive expression in the *cVg1*-expressing side in isolated anterior halves (Figure 8).

**Figure 7—figure supplement 1.**
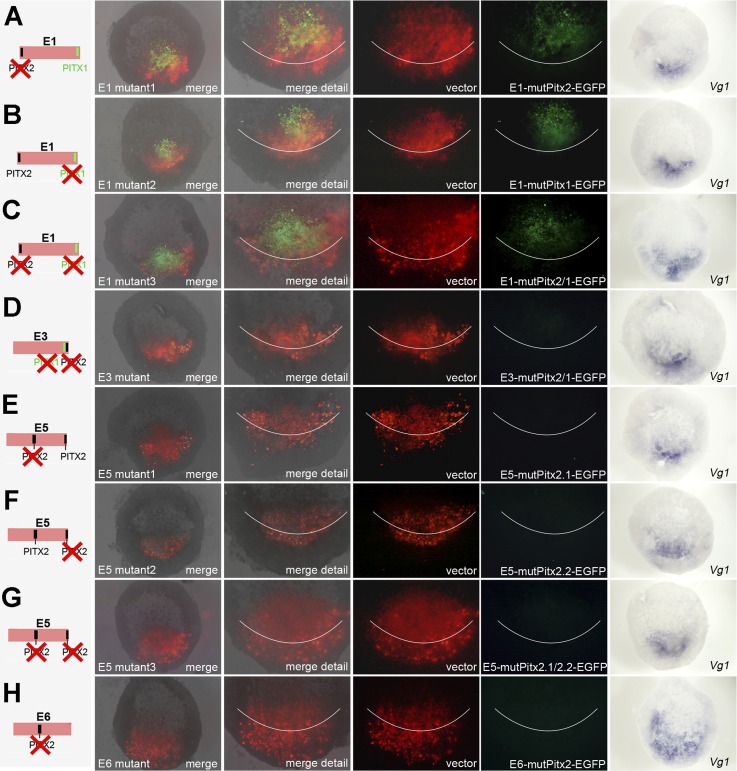
Effects of mutations in Pitx-binding sites on activity of cVg1 enhancers in whole embryos. Each of the enhancers containing Pitx1- or Pitx2-binding sites was mutated either singly or, for those enhancers containing more than one discrete site, both together. The mutation is shown diagrammatically on the left column. In columns 2–5, the area electroporated (DS-RedExpress reporter) appears in red and expression driven by the specific enhancer construct in green. The last column shows *cVg1* expression revealed by in situ hybridisation in the same embryos. Enhancer E1 is unaffected by mutation of either Pitx1- or Pitx2-binding sites, and continues to be active inside the embryo but not in the PMZ. E6 is also unchanged by mutation of the Pitx2-binding site (no expression is seen from the intact reporter, see main Figure 7). The activity of enhancers and E3, E5 is destroyed by mutation of any of the Pitx1/2-binding sites. **DOI:** http://dx.doi.org/10.7554/eLife.03743.020

**Figure 8—figure supplement 1.**
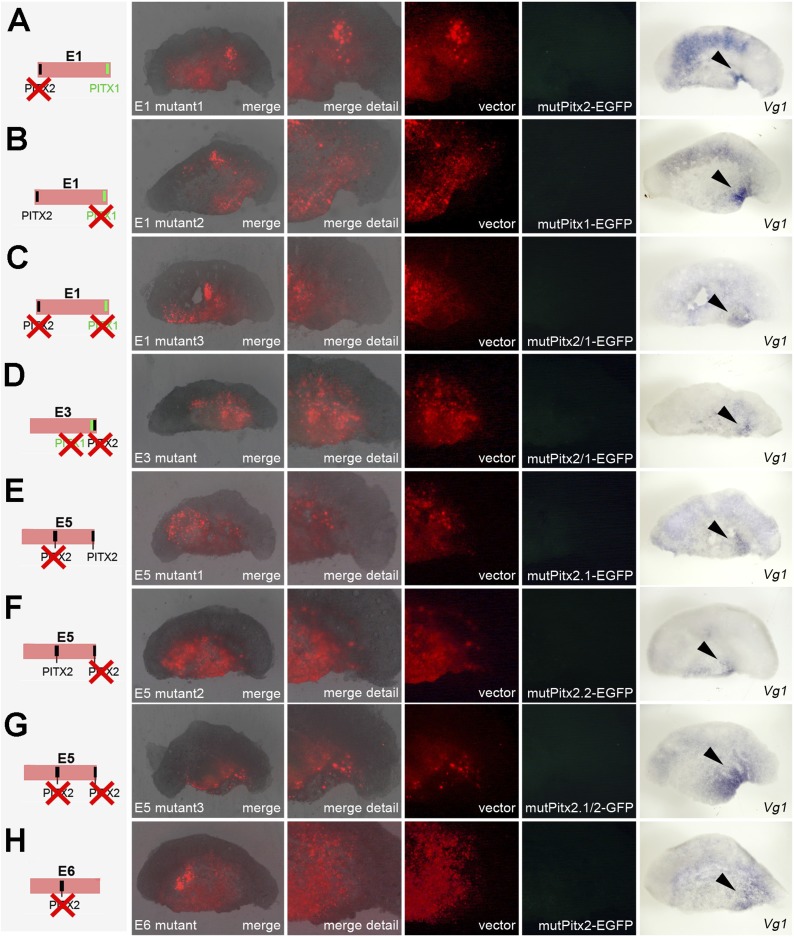
Effects of mutations in Pitx-binding sites on activity of *cVg1* enhancers in isolated anterior halves. Each of the enhancers containing Pitx1- or Pitx2-binding sites was mutated either singly or, for those enhancers containing more than one discrete site, both together. The mutation is shown diagrammatically on the left column. Details as in Figure 7—figure supplement 1. The specific activity of E3 and E5 is destroyed by mutation of any of the Pitx-binding sites. **DOI:** http://dx.doi.org/10.7554/eLife.03743.022

To test whether the Pitx-binding sites are required within these enhancers to direct expression to the appropriate domain, we generated reporter constructs for enhancers E1, E3, E5, and E6 containing mutations in each Pitx1- or Pitx2-binding site as well as constructs where all Pitx-binding sites were mutated. In whole embryos, mutations in either of the Pitx-binding sites (Figure 7—figure supplement 1A–B) or in both binding sites (Figure 7—figure supplement 1C) of E1 still showed GFP expression without selectivity, in the middle of the embryo. For E3 and E5, however, mutations in either a single or both binding sites of each reporter completely abolished expression in the PMZ (Figure 7—figure supplement 1D–G). Un-mutated reporter E6, which did not show any activity (see above), was not altered by a mutation of its Pitx2-binding site (Figure 7—figure supplement 1H). Similar results were found in isolated anterior half-embryos: mutations of any of the Pitx-binding sites in E3 or E5 completely eliminated expression in the *cVg1*-expressing corner of the isolated half (Figure 8—figure supplement 1).

Taken together, these results suggest that *cVg1* expression is regulated directly by Pitx2/Pitx1 binding to an enhancer (E5) within an intron of the *HOMER3* gene, adjacent to *cVg1*/*GDF3*, and to an intronic enhancer (E3) within the *Lass1*/*CERS1* locus. The ChIP experiments suggest that Pitx2 binding to an additional intronic enhancer in *HOMER3* (E6) may also be functional in the PMZ, although this was not seen when a reporter construct containing this element was electroporated into the PMZ. The same enhancers (E3 and E5) are involved in controlling *cVg1* expression in the normal PMZ as in the portion of the lateral marginal zone where *cVg1* is upregulated a few hours after isolation of a portion of embryo. These results implicate the transcription factor Pitx2 as the earliest gene described to date that regulates the position of the embryonic axis as well as embryonic regulation/regeneration and twinning.

## Discussion

The PMZ of the chick embryo is the equivalent of the Nieuwkoop centre of amphibians: it can induce a complete axis including the organiser from neighbouring cells, without making a cellular contribution to the axis (Azar and Eyal-Giladi, 1979; Khaner and Eyal-Giladi, 1989; Bachvarova et al., 1998). The Nodal/Activin-related TGFβ superfamily member *cVg1* (homologous to mammalian *GDF1*) is expressed in the PMZ. When ectopically applied to another region of the marginal zone, *cVg1* is sufficient to initiate formation of a complete embryonic axis from adjacent embryonic (area pellucida) cells (Seleiro et al., 1996; Shah et al., 1997). To act, cVg1 requires canonical Wnt, which seems to be provided mainly by *cWnt8C*, expressed all around the marginal zone (Skromne and Stern, 2001). A target of cVg1 and Wnt is *Nodal*, transcribed in area pellucida cells next to the *cVg1*+*Wnt* expression domain (Skromne and Stern, 2002). The anterior end of the embryo also has an early identity, defined by the expression of GATA binding protein 2 (GATA2) (Sheng and Stern, 1999; Bertocchini and Stern, 2012). However, unlike cVg1, GATA2 is not a sufficient determinant of polarity and at best only acts as a bias (Bertocchini and Stern, 2012).

While amphibian and teleost embryos lose their ability to generate complete, independent embryos if fragmented after the first few cell divisions, amniotes have huge regulative capacity. Dividing a chick blastoderm even just before primitive streak formation (when the embryo may have as many as 50,000 cells) into up to eight fragments can lead to formation of as many complete embryos (Lutz, 1949; Spratt and Haas, 1960). In an isolated anterior half, a visible primitive streak (and expression of *Brachyury* and *Snail2*) can be detected about 12 hr after cutting. This appears randomly from either the left or right area pellucida adjacent to the cut edge of the marginal zone (Spratt and Haas, 1960), preceded at least 6 hr earlier by expression of *cVg1* in the right or left marginal zone (Bertocchini et al., 2004). Blocking cVg1 on one side of the marginal zone of an isolated anterior half will cause the axis to arise from the opposite side. A similar manipulation in the PMZ will cause a streak to arise from outside the MO-electroporated domain (Bertocchini et al., 2004; Bertocchini and Stern, 2012). Thus, *cVg1* expression in the marginal zone is both necessary and sufficient to initiate formation of a primitive streak.

There was no information, however, about what positions *cVg1* expression in the PMZ of normal embryos or in isolated fragments. This led us to undertake a screen for upstream regulators. We took two complementary approaches: a molecular screen, designed to identify genes co-expressed with *cVg1* both in the normal PMZ and in the *cVg1*-expressing edge of the marginal zone in isolated anterior halves at the time when *cVg1* first becomes detectable, and a bioinformatics-based approach, predicting and analysing putative enhancers of *cVg1* situated on chromosome 28. A particular difficulty of the molecular screen was that it is impossible to know, in a single embryo, which of the two edges of the isolated anterior half will express *cVg1*. This required analysis of each individual fragment just after excising left and right edge explants, then pooling them appropriately according to whether they derived from the *cVg1*-expressing side or the opposite edge. This has the additional advantage of removing any intrinsic left–right differences that may exist in the embryo at this stage. Selecting genes that are co-expressed with *cVg1* when this is first upregulated at one edge of an isolated fragment, and also co-expressed with *cVg1* in the normal PMZ, turned out to be a powerful strategy to reduce the number of relevant genes, thereby avoiding the ‘cherry-picking’ approaches often associated with molecular screens. This combination of molecular screens and bioinformatic analysis converged towards a single strong candidate: the transcription factor *Pitx2*. The regulatory role of Pitx2 on *cVg1* expression was then confirmed by loss-of-function experiments showing that Pitx2 is required for *cVg1* activation and axis formation both in normal development and during embryonic regulation, and that it binds directly to four non-coding regions around the *cVg1* locus (E1, E3, E5, and E6), two of which (E3 and E5) are sufficient to direct expression specifically to the PMZ and *cVg1*-expressing edge of an isolated anterior fragment. Moreover, mutation of any of the Pitx-binding sites in enhancers E3 and E5 abolishes the activity of these enhancers in the PMZ. These findings strongly implicate Pitx2 as a direct and essential regulator of *cVg1* expression.

Pitx transcription factors are characterised by possessing a Lysine residue at position 50 of the homeodomain, an unusual property shared with the vertebrate genes *Goosecoid* and *OTX-1/2* and with the founder gene of the family, Drosophila *Bicoid*. In Drosophila, Bicoid is a critical specifier of ‘anterior’ identity and essential for setting up head–tail polarity of the early embryo. Although it is tempting to speculate that a *Bicoid*/*Pitx* system may have an ancient function in the specification of head–tail polarity, the *Bicoid* gene does not appear to have direct orthologues in species other than schizophoran flies (closely related to Drosophila), so this may be either a coincidence or convergent evolution.

The four key components that form part of this gene regulatory network initiating axis formation, *Pitx2*, *cVg1/GDF1*, *Tbx6,* and *Nodal*, are also involved a little later in development (from the late primitive streak stage) in specifying left–right asymmetry in different vertebrate classes (Levin et al., 1995; Hyatt et al., 1996; Hyatt and Yost, 1998; Logan et al., 1998; Meno et al., 1998; Piedra et al., 1998; Ryan et al., 1998; St Amand et al., 1998; Yoshioka et al., 1998; Zhu et al., 1999; Rankin et al., 2000; Wall et al., 2000; Levin, 2005; Raya and Izpisua Belmonte, 2006; Tanaka et al., 2007; Hadjantonakis et al., 2008). Indeed, these are the main conserved components of the left–right pathway among different vertebrates. These observations raise the possibility that the left–right pathway may have evolved by co-opting a more ancient mechanism for initiating formation of the gastrular axis. This is supported by the finding that a *Nodal*/*Pitx2* loop is involved in specifying both left–right asymmetry and mesendoderm formation (oral-aboral polarity) in the sea urchin, a non-vertebrate deuterostome (Duboc et al., 2004; Hibino et al., 2006; Warner et al., 2012). *Pitx2*, *Vg1*, *Nodal,* and *Tbx6* are also involved in early mesendoderm development in anamniotes, although *Vg1* is maternal (Thomsen and Melton, 1993; Kessler and Melton, 1995; Faucourt et al., 2001).

In the mouse, double mutants for Pitx1 and Pitx2 lead to early lethality (at pre- or peri-implantation stages) of the embryo (Marcil et al., 2003). Only a single embryo was ever recovered that had survived to E10–E12 (Drouin, personal communication). Expression of these transcription factors has to date only been studied in detail at later stages of mouse development (L'Honore et al., 2007; Lanctot et al., 1997) and it will therefore be interesting to see if they are indeed expressed as in the chick at pre-primitive streak stages. Certainly, the strong conservation of the active enhancers (E3 and E5, including the Pitx-binding sites contained therein) near mouse and human GDF1 suggests that this is likely to be a conserved feature of birds and mammals, and despite the fact that the peculiar geometry of rodent embryos at these early stages has led to some differences in the processes leading to axis development (Stern and Downs, 2012). Monozygotic twins do not seem to occur commonly in the mouse and it is possible that these very small, cup-shaped embryos do not survive to term if more than one primitive streak appears within a single blastocyst. Together, these findings suggest that the retention of a regulative mode of development at late stages by amniote embryos (that allows the formation of the types of monozygotic twins that arise relatively late in development, including Siamese twins) evolved through novel uses of an ancient pathway, involved in both mesendoderm development and left–right asymmetry, but in slightly different ways.

Our study also brings forward the time at which the earliest responses to cutting an embryo can be detected. First, we can now detect *cVg1* expression at one edge of a cut anterior fragment 4–5 hr after cutting, about 2 hr earlier than the 6 hr reported previously (Bertocchini et al., 2004); *Pitx2* appears even earlier, 3 hr after cutting. But this also begs the question of what lies upstream of *Pitx2*. At some early point in the cascade, the regulators will no longer be controlled at the transcriptional level and it will be considerably more difficult to identify them. The present finding that *Pitx2* is upregulated locally just 3 hr after cutting an embryo suggests that its regulators may not be differentially expressed mRNAs, but other asymmetries. Our studies allow us to predict the *Pitx2*/*cVg1*(*GDF1*)/*Nodal* pathway as a possible candidate to explain the obligate quadruplets of armadillos (Newman and Patterson, 1910; Loughry et al., 1998; Enders, 2002; Eakin and Behringer, 2004) and/or the high incidence of monozygotic and conjoined twins in certain human populations (Cox, 1963; Hamamy et al., 2004; Forsberg et al., 2010). Answering these questions represent interesting future challenges.

## Materials and methods

### Embryos, manipulation and RNA in situ hybridisation

Fertile Brown Bovan Gold hens' eggs (Henry Stewart, UK) were incubated for 1–16 hr to obtain stages X–XIII (Eyal-Giladi and Kochav, 1976) and stage 4 (Hamburger and Hamilton, 1951) (HH). Embryo manipulation was performed in Tyrode's solution. Anterior halves were obtained by cutting embryos with a hair loop. Unlike previous studies (Bertocchini et al., 2004), here we did not use a strip of anterior area opaca to seal the cut edge. Embryos and fragments were set up in modified New culture (New, 1955; Stern and Ireland, 1981) and incubated at 38°C as required. Whole mount in situ hybridisation was performed as described previously (Stern, 1998; Streit and Stern, 2001). The probes used were: chick *cVg1* (Shah et al., 1997), *Brachyury* (Kispert et al., 1995a; Kispert et al., 1995b; Knezevic et al., 1997), and *Pitx2* (Logan et al., 1998; Zhu et al., 1999).

### Microarray screens, analysis and verification of candidate genes

Two microarray screens were performed with tissues collected from stages X–XII (Eyal-Giladi and Kochav, 1976). A first screen was performed with triplicates of 40 pieces of PMZ and AMZ, dissected and individually frozen from whole embryos (Figure 1A, Figure 1—figure supplement 1). A second screen was done with triplicates of 70 pieces of the left and right corners of the marginal zone, dissected and frozen individually from anterior embryo halves that had been cultured for 7 hr (Figure 1C, Figure 1—figure supplement 2). After dissection, whole embryos and anterior halves were fixed in 4% PFA for in situ hybridisation with *cVg1*. In both screens, in situ hybridisation was carried out for an extended period to detect low *cVg1* expression adjacent to the excised pieces and confirm the orientation of the embryo (see Results); the orientation was ambiguous or incorrect in about 10% of the embryos, and explants obtained from them were therefore not included. The remaining validated PMZ and AMZ samples, *cVg1*-like and non-*cVg1*-like samples were pooled in TRIzol reagent (Ambion, Invitrogen, UK). RNA was prepared and run for a complete Affymetrix analysis by ARK-Genomics. 500 ng of total RNA was required for the standard 3' IVT-Express protocol. Each label was quality checked through all stages of amplification and preparation for hybridisation on Affymetrix 30K chicken microarrays. Microarray raw data were analysed using Bioconductor in R (Gentleman et al., 2004). Raw datasets were normalised using the Robust Multi-array Average (RMA) method (Irizarry et al., 2003). Differentially expressed genes were then identified using the Limma package in R (Smyth, 2005) with a fold change threshold of 1.2 and p < 0.05. This strategy identified 122 sequences (corresponding to 85 genes) with putative *cVg1*-like expression (‘*cVg1*-like’) and 78 sequences (52 genes) expressed in the *cVg1*-negative explants (‘*cVg1*-unlike’). The complete dataset was deposited with ArrayExpress where it can be accessed under Accession number E-MTAB-3116.

A selection of genes from the top up- and downregulated genes was verified by in situ hybridisation: *ADMP*, *PITX2*, *THPO*, *PKDCC*, *PMEPA*, *TBX6*, *FGF8*, *Ovoinhibitor*, *ELK3*, *LITAF*, *PITX1*, *CAMK1G*, *DENND5B*, *MLLT6*, *Homer3*, *SEMA5B*, *PIK3R1*, *GALNTL1*, *SH2D4A*, *GLI3*, *PDLIM5*, *VANGL1*, *LEF1*, *FGF18*, *LOC768709*, *CXCL14*, *PLCB1*, *ZBTB1*, *MED15*, *SPOCK3*, *BASP1*, *LRP2*, *GABRB2*, *PDGFA*, *Autotaxin*, *SALL1*, *MAFA*, *ESRRG*, *FOXD2*, *FSTL4*, *MALT1*, *OLFM3*, *GNAZ* and *GRB10*; when not already available, probes were generated from the chick EST collection (Boardman et al., 2002).

### Insulator analysis

Computational prediction of CTCF insulator elements was performed as previously described (Khan et al., 2013). A PERL script (http://www.ncbi.nlm.nih.gov/pmc/articles/PMC3664090/#SD1) was used to scan chromosome 28 (location of *GDF3*/*cVg1*) from the galGal4 build of the chicken genome for occurrences of CTCF-binding sites with stringent parameters (False Discovery Rate, FDR 0%). Equivalent regions of human chromosome 19 (hg19 genome build, location of homologous GDF1) and mouse chromosome 8 (mm10 genome build, location of homologous GDF1) were also scanned for CTCF-binding sites with the same FDR parameter. The nearest conserved CTCF-binding sites harbouring the same set of genes both upstream and downstream of GDF3/GDF1 in all three species were then identified and these domains were defined as regions bounded by putative insulators.

### Enhancer prediction

Enhancer prediction was carried out using the software package Discovery of Regulatory Elements in Vertebrates (DREiVe) (Khan et al., 2013), the vertebrate version of EDGI (Sosinsky et al., 2007). Genomic coordinates for the predicted insulator region in human were used as the reference to predict order-independent conserved patterns of DNA sequences shared between human and any seven of the following species: horse (Equcab2), cow (Bostau4), rabbit (Orycun2), guinea pig (Cavpor3), mouse (mm9), opossum (Mondom5), platypus (Taegut1), chicken (Galgal3), and lizard (Anocar1). Parameters used included motif density of 6 matching nucleotides within a window length of 8 bp, where the minimum number of matching nucleotides in the motifs was set at 12 bp. The maximal cluster length (maximum length of predicted enhancers) was set at 3000 bp with a sequence conservation score cut-off of 2. This set of parameters successfully predicted a series of conserved blocks, designated E1–E4 (see Figure 5), in human, chicken, mouse, and other species. Transcription factor binding site analysis of these predicted enhancers was carried out using the matrix-scan algorithm from the RSAT workbench (http://rsat.ulb.ac.be/rsat/) (Thomas-Chollier et al., 2011). Position frequency matrices from both Jaspar (http://jaspar.cgb.ki.se/) and Transfac (http://www.gene-regulation.com/pub/databases.html) libraries were used in matrix-scan where the background model estimation method was based on a Markov order of 0. Organism-specific ‘upstream no-orf’ background sequences were used (galGal4) with a pseudo-frequency of 0.01 and an upper p-value threshold of 1e^−4^. As a complementary approach, a modified methodology of Clover (http://cagt.bu.edu/page/Clover_about) was used to detect enhancers that shared order-independent transcription factor binding sites rather than DNA patterns. This approach uncovered two additional putative enhancers, E5 and E6.

### Electroporation, morpholinos and DNA constructs

Fluorescein-labelled MOs against *Pitx2*, *Pitx1* and a standard control-MO (Gene Tools, Philomath, Oregon, USA) were delivered to young embryos by electroporation as described (Voiculescu et al., 2007; Voiculescu et al., 2008). *Pitx2*-MO was designed to target the translational start site: (5′-CAAGTTTACGGCAGTTGGACTCCAT-3′). A splice blocking *Pitx1*-MO was designed to target the start of exon-2: (5′-CTCTCTTTTTCTACGGTGGGATGTT-3′). The coding sequence of chicken *Pitx2* (Logan et al., 1998) was cloned into pCAβ-IRES-GFP to generate a *Pitx2* expression construct. The expression of *Pitx2* protein was confirmed by western blot with a Pitx2-antibody (Abcam, UK, ab55599). Candidate enhancers 1–6 (E1–E6) were amplified from chick genomic DNA using the following primer pairs: (E1)-forward (5′-CAGCCCCAGGCAGACAGGGCTGCAGGGAAGAAGGGG-3′), reverse (5′-ACGGGACCCCCAGCCCTGCAGGATGCTGCCCGGGGT-3′); (E2)-forward (5′-GGAGGTACCATAATTCATGCTTTCTGGGCTCGGGAC-3′), reverse (5′-GTACTCGAGCAAAGGTATCCCAGACCCTGCTGTC-3′); (E3)-forward (5′- GGAGGTACCACTCATTTGGTTTTAGCATTAATAAAC-3′), reverse (5′- GTACTCGAGCTGCCAGGGCAGAGGGAGCAGGGTG-3′); (E4)-forward (5′-CAGGGGATGAAGGGGGTGTTGGGGATCAAGCTCTTC-3′), reverse (5′-CAGTCTGCTACAATCCCTTCCCATGGATTCCTGGGG -3′); (E5)-forward (5′-GTGAGGTACCCTGTTCAGTC-3′), reverse (5′- AACTCGAGGTACAAGCTCTGC -3′); (E6)-forward (5′-GGTAGAGACCTGGTACCAGTAG-3′), reverse (5′-GAAGGGAGCTCGAGTGTCAC -3′). The amplified fragments were subcloned into pGEMT-easy (Promega, Madison, Wisconsin, USA), excised with inserted *KpnI* and *XhoI* sites and cloned into ptkEGFP (Uchikawa et al., 2003) or ptkRFP (kind gifts of H Kondoh). All constructs were checked by sequencing. 3 µg of each enhancer construct were co-electroporated with pCAβ-RFP, DS-RedExpress (Invitrogen, UK) or pCAβ-IRES-GFP as appropriate.

Mutations were introduced into each of the Pitx1- or Pitx2-binding sites of the four enhancers (E1, E3, E5, and E6) that contained such sites, with base changes highlighted in red (Table 5). The PCR primers used for site-directed mutagenesis are shown in Table 6. For Enh5, site 1 refers to the 5′ Pitx2, while site 2 is the 3′ Pitx2-binding site (see Figure 5). Site-directed mutagenesis was performed as described (Liu and Naismith, 2008). Briefly, 100 ng of template Enhancer DNA was PCR amplified with 2 µM of each mutant primer pair, 200 µM dNTPs, 2 µl Phusion high-fidelity polymerase, and 5 µl 10X buffer in a total volume of 50 µl. The PCR programme was as follows: 94°C 3 min, 94°C 1 min, 52°C 1 min, 68°C 8, 12, or 24 min (500 bp/min), final extension 68°C 1 hr, then 4°C. 1/25th of each reaction was run on an Agarose gel to verify amplification, after which the remainder of the reaction was digested with DpnI to remove the parent template for 1 hr at 37°C. Another 1/25th of the DpnI digest was transformed into DH5α cells and clones selected and amplified in culture for DNA extraction and sequence verification of the introduced mutations.

**Table 5.** Construction of mutations introduced to destroy Pitx-binding sites **DOI:** http://dx.doi.org/10.7554/eLife.03743.023

Enh PITX site	Mutated bases
E1 PITX2	GCTGGTCATGACTTCTT
E1 PITX1	TGGTTTCGAGTTGAAAG
E3 PITX2	GGGTGTCATGAATGGCC
E3 PITX1	AGGGTTCGAGTAATGGC
E5 PITX2 1	TAAAATCCTGAAACTGC
E5 PITX2 2	AGAAATCATGAATAAAT
E6 PITX2	TGTCTTCATGACTCCTC

The mutations (highlighted in red) introduced into the sequences of Pitx-binding sites in enhancers E1, E3, E5 and E6.

**Table 6.** Primers used for construction of mutations in Pitx-binding sites **DOI:** http://dx.doi.org/10.7554/eLife.03743.024

Primer sequence	Primer name
aggggtttggttTCgaGttgaaagcgtgtacttctcacccattgaaactgccaggtctgt	Enh1PitX1MutF
ctttcaaCtcGAaaccaaacccctcccatcacacacacaaactctactgcttcttcaaacca	Enh1PitX1MutR
agggggctggTCatGacttcttgcaggtgccccaaaggcagggcca	Enh1PitX2MutF
aagaagtCatGAccagccccctacccgaccctgctgcacagggaat	Enh1PitX2MutR
ggccattaCtcGAaccctgctccctctgccctggcag	Enh3PitX2MutF
ctgccagggcagagggagcagggtTCgaGtaatggcc	Enh3PitX2MutR
agtgcagtttCagGAttttactttatagtttttttattcctacttgccatgaaagtggagaacat	Enh5PitX2MutF
taaaaTCctGaaactgcacttaacatagcgattacaaaacactcttggttagagtagcaacacaa	Enh5PitX2MutR
tgggctttcctatttattCatGAtttcttacagtagacaagaagattcacatttgtttgtgtttcatagctagag	Enh5PitX22MutF
tgtaagaaaTCatGaataaataggaaagcccaaatgttacaagcttcattgggcctccattgctggaagaaaaga	Enh5PitX22MutR
ataacaaaggggaggagtCatGAagacattgattctaatttgttctacatttcagtttattagcaaagtgaca	Enh6PitX2MutF
aatcaatgtctTCatGactcctcccctttgttatttcattttatgtgctttctaattcatcttcaattaaga	Enh6PitX2MutR

This table shows the primer combinations used to generate the mutations indicated in Table 5.

### ChIP and qPCR

Micro ChIP experiments were performed with pools of 25 pieces of PMZ and their anterior counterparts (AMZ) from whole pre-streak stage X–XI embryos. Chromatin was cross-linked, extracted and sonicated to obtain 200–1000 bp fragments. Immunoprecipitation was done with rabbit anti-H3K27ac (Abcam, UK), mouse anti-PITX2 (Abcam, ab55599), antibodies and rabbit IgG (Millipore, Merck Millipore UK) and mouse IgG (Millipore, Merck Millipore UK) bound to magnetic Dynabeads (Life Technologies, Carlsbad, California, USA). The purified DNA was used as template for qPCR analyses with a Bio-Rad iCycler and Sybr Fast Q-PCR mix (Kapa Biosystems, Wilmington, Massachusetts, USA). The following primer pairs were used: enhancer1—forward primer1 E1F1 (5′-gctctatcccgattccctgtgca-3′) reverse primer1 E1R1 (5′- ggtagggttcacttcattagggatg -3′, forward primer2 E1F5 (5′-cactctgtgtccggagaatgctac-3′) reverse primer2 E1R5 (5′- caatgggtgagaagtacacgctttc-3′ ; enhancer2—forward primer1 E2F1 (5′- atgaagagcgcaagagtggcaaag-3′) reverse primer1 E2R1 (5′-tgggaaattccgacctggaagcag-3′, forward primer2 E2F3 (5′- agctgagctgtttaacggtggatc-3′) reverse primer2 E2R3 (5′- agctgaccgctgctagttctct-3′); enhancer 3—forward primer E3F2 (5′- cctagctattacactctgctcttcc-3′) reverse primer E3R2 (5′- CAGTAGAGACGGAACCAGAACct-3′); enhancer 4—forward primer1 E4F1 (5′- gcttcttggtgctggaactgagaat-3′) reverse primer1 E4R1(5′- atggagagacacagtcagccacga-3′, forward primer2 E4F2 (5′- agctgcccaatgctctgaaagaag -3′) reverse primer2 E4R2 (5′- ggaatggaaaggtgaggattcatgg -3′); enhancer 5—forward primer E5F3 (5′- CGTGAGGCAGTCTGTTCAGT-3′) reverse primer E5R3 (5′- GCTATGAAACACAAACAAATGTGAA-3′); enhancer 6—forward primer E6F3 (5′- CACTGGGGTCCTGATGTAGTG-3′), reverse primer E6R3 (5′- AGAAGGGAGCACAAATGTCA-3′); Vg1 -0.5 ATG—forward primer Vg1F1 (5′-agtgggtgctgattgctgtctgtg-3′) reverse primer Vg1R1 (5′-ctcccatcccttctgcatctccata-3′). All qPCR reactions were run in triplicate and contained an input control for normalisation of chromatin concentration, and the appropriate IgG as control. The amplification products were quantified using the ΔΔCt method (Livak and Schmittgen, 2001).

## Additional files

### Major dataset

The following dataset was generated:

Khan M, Torlopp A, Stern CD, 2014, Pitx2 positions the embryonic axis and regulates twinning; Publicly available at ArrayExpress (https://www.ebi.ac.uk/arrayexpress/).
