# Supplementary material for: The transcription factor Pitx2 positions the embryonic axis and regulates twinning
Source: eLife. 2014 Dec 12;3:e03743. doi: 10.7554/eLife.03743 (PMC4371885; doi:10.7554/eLife.03743)
Supplement: Figure 4—source data 1. — Each type of experiment is summarised in a separate sub-table, where each row refers to a particular column (A–J) and row (first, second, and third) of panels in Figure 4. The table columns show each type of result obtained: ‘Vg1 R’ refers to embryos showing cVg1 expression on the right side, ‘streak L’ when there is a Brachyury-expressing primitive streak on the left, ‘streak L/R’ when there are two streaks (one arising from each side), etc. DOI: http://dx.doi.org/10.7554/eLife.03743.014 [file elife03743s001.docx]

**Source data for Figure 4**

Table 1a: Pitx2 loss of function in half embryos after 7hrs

| **FIGURE 4** | **MORPHOLINO** | **Vg1 R** | **Vg1 L** | **Vg1 L/R** | **NO Vg1** | **total** |
| --- | --- | --- | --- | --- | --- | --- |
| **A (1st row)** | **Ctrl MO - R** | **2 (33%)** | **2 (33%)** | **-** | **2 (33%)** | **6** |
| **B (1st row)** | **Pitx2 MO - R** | **-** | **1 (10%)** | **-** | **9 (90%)** | **10** |
| **not shown** | **Pitx2 MO - R after 9hrs** | **-** | **↑2 (33%)** | **-** | **↓4 (66%)** | **6** |
| **C (1st row)** | **Ctrl MO - L** | **1 (20%)** | **3 (60%)** | **-** | **1 (20%)** | **5** |
| **D (1st row)** | **Pitx2 MO - L** | **1 (17%)** | **-** | **2 (33%) [2R]** | **3 (50%)** | **6** |
| **not shown** | **Pitx2 MO - L after 9hrs** | **↑3 (60%)** | **-** | **-** | **↓2 (40%)** | **5** |

Table 1b: Pitx2 loss of function in half embryos after 16hrs

| **FIGURE 4** | **MORPHOLINO** | **streak R** | **streak L** | **streak L/R** | **NO streak** | **total** |
| --- | --- | --- | --- | --- | --- | --- |
| **A (2nd row)** | **Ctrl MO - R** | **3 (30%)** | **3 (30%)** | **2 (20%) [1R,1L]** | **2 (20%)** | **10** |
| **B (2nd row)** | **Pitx2 MO - R** | **-** | **8 (80%)** | **2 (20%) [2R/L]** | **-** | **10** |
| **C (2nd row)** | **Ctrl MO - L** | **4 (33%)** | **4 (33%)** | **3 (25%) [1R,2R/L]** | **1 (9%)** | **12** |
| **D (2nd row)** | **Pitx2 MO - L** | **2 (25%)** | **-** | **1 (12,5%) [1R]** | **5 (62,5%)** | **8** |

Table 1c: Pitx2 loss of function in whole embryos after 5hrs

| **FIGURE 4** | **MORPHOLINO** | **normal Vg1 expr** | **loss of Vg1 expr** | **total** |
| --- | --- | --- | --- | --- |
| **E (1st row)** | **Ctrl MO** | **7 (100%)** | **-** | **7** |
| **F (1st row)** | **Pitx2 MO** | **-** | **6 (100%)** | **6** |

Table 1d: Pitx2 loss of function in whole embryos after 12 and 16hrs

| **FIGURE 4** | **MORPHOLINO** | **normal streak** | **displaced streak** | **double streak** | **loss of streak** | **total** |
| --- | --- | --- | --- | --- | --- | --- |
| **E (2nd row)** | **Ctrl MO > 12hrs** | **4 (100%)** | **-** | **-** | **-** | **4** |
| **F (2nd row)** | **Pitx2 MO > 12hrs** | **3 (25%)** | **3** | **3** | **3** | **12** |
| **E (3rd row)** | **Ctrl MO > 16hrs** | **4 (100%)** | **-** | **-** | **-** | **4** |
| **F (3rd row)** | **Pitx2 MO > 16hrs** | **7 (88%)** | **1 (22%)** | **-** | **-** | **8** |

Table 1e: Inhibition of both Pitx2 and Pitx1 in whole embryos after 5hrs

| **FIGURE 4** | **MORPHOLINO** | **normal Vg1 expr** | **loss of Vg1 expr** | **total** |
| --- | --- | --- | --- | --- |
| **E (1st row)** | **Ctrl MO** | **4 (100%)** | **-** | **4** |
| **G (1st row)** | **Pitx1 MO + Pitx2 MO** | **-** | **6 (100%)** | **6** |

Table 1f: Inhibition of both Pitx2 and Pitx1 in whole embryos after 12 and 16hrs

| **FIGURE 4** | **MORPHOLINO** | **normal streak** | **displaced streak** | **double streak** | **loss of streak** | **total** |
| --- | --- | --- | --- | --- | --- | --- |
| **E (2nd row)** | **Ctrl MO > 12hrs** | **4 (100%)** | **-** | **-** | **-** | **4** |
| **G (2nd row)** | **Pitx1 MO + Pitx2 MO > 12hrs** | **1 (11%)** | **3** | **3** | **2** | **9** |
| **E (3rd row)** | **Ctrl MO >16hrs** | **4 (100%)** | **-** | **-** | **-** | **4** |
| **G (3rd row)** | **Pitx1 MO + Pitx2 MO > 16hrs** | **-** | **-** | **4 (100%)** | **-** | **4** |

Table 1g: Pitx2 rescue in whole embryos after 5hrs

| **FIGURE 4** |  | **normal Vg1 expr** | **displaced Vg1 expr** | **total** |
| --- | --- | --- | --- | --- |
| **E (1st row)** | **Control MO** | **4 (100%)** | **-** | **4** |
| **H (1st row)** | **Pitx1 MO + Pitx2 MO + Pitx2 Rescue** | **5 (83%)** | **1 (17%)** | **6** |

Table 1h: Pitx2 rescue in whole embryos after 12 and 16hrs

| **FIGURE 4** |  | **normal streak** | **displaced streak** | **double streak** | **loss of streak** | **total** |
| --- | --- | --- | --- | --- | --- | --- |
| **E (2nd row)** | **Control MO > 12hrs** | **4 (100%)** | **-** | **-** | **-** | **4** |
| **H (2nd row)** | **Pitx1 MO + Pitx2 MO + Pitx2 Rescue > 12hrs** | **8 (80%)** | **-** | **2 (20)** | **-** | **10** |
| **E (3rd row)** | **Control MO > 16hrs** | **4 (100%)** | **-** | **-** | **-** | **4** |
| **H (3rd row)** | **Pitx1 MO + Pitx2 MO + Pitx2 Rescue > 16hrs** | **12 (75%)** | **-** | **4 (25%)** | **-** | **16** |

Table 1i: Inhibition of both Pitx2 and Pitx1 in half embryos after 7 hrs

| **FIGURE 4** | **MORPHOLINO** | **Vg1 R** | **Vg1 L** | **Vg1 L/R** | **NO Vg1** | **total** |
| --- | --- | --- | --- | --- | --- | --- |
| **A (1st row)** | **Ctrl MO - R** | **2 (33%)** | **2 (33%)** | **-** | **2 (33%)** | **6** |
| **I – 7hrs** | **Pitx1 MO + Pitx2 MO - R** | **-** | **-** | **-** | **7 (100%)** | **7** |

Table 1j: Inhibition of both Pitx2 and Pitx1 in half embryos after 16hrs

| **FIGURE 4** | **MORPHOLINO** | **streak R** | **streak L** | **streak L/R** | **no streak** | **total** |
| --- | --- | --- | --- | --- | --- | --- |
| **A (2nd row)** | **Ctrl MO - R** | **3 (30%)** | **3 (30%)** | **2 (20%) [1R,1L]** | **2 (20%)** | **10** |
| **I – 16hrs** | **Pitx1 MO + Pitx2 MO - R** | **-** | **5 (100%)** | **-** | **-** | **5** |

Table 1k: Pitx2 rescue in half embryos after 7hrs

| **FIGURE 4** |  | **Vg1 R** | **Vg1 L** | **Vg1 L/R** | **NO Vg1** | **total** |
| --- | --- | --- | --- | --- | --- | --- |
| **not shown** | **Control MO** | **3 (60%)** | **2 (40%)** | **-** | **-** | **5** |
| **J – 7hrs** | **Pitx1 MO + Pitx2 MO + Pitx2 Rescue** | **4 (40%)** | **3 (30%)** |  | **3 (30%)** | **10** |

Table 1l: Pitx2 rescue in half embryos after 16hrs

| **FIGURE 4** |  | **streak R** | **streak L** | **streak L/R** | **no streak** | **total** |
| --- | --- | --- | --- | --- | --- | --- |
| **not shown** | **Control MO** | **3 (60%)** | **2 (40%)** | **-** | **-** | **5** |
| **J – 16hrs** | **Pitx1 MO + Pitx2 MO + Pitx2 Rescue** | **3 (38%)** | **2 (24%)** |  | **3 (38%)** | **8** |
